# Alleviating arsenic stress affecting the growth of *Vigna radiata* through the application of *Klebsiella* strain ASBT-KP1 isolated from wastewater

**DOI:** 10.3389/fmicb.2024.1484069

**Published:** 2024-09-25

**Authors:** Megha Prasad, Ajith Madhavan, Pradeesh Babu, Amrita Salim, Suja Subhash, Bipin G. Nair, Sanjay Pal

**Affiliations:** School of Biotechnology, Amrita Vishwa Vidyapeetham, Kollam, Kerala, India

**Keywords:** plant growth-promoting bacteria, arsenic tolerance, bioremediation, *Vigna radiata*, whole genome sequencing

## Abstract

Arsenic contamination of soil and water is a major environmental issue. Bioremediation through plant growth-promoting bacteria is viable, cost-effective, and sustainable. Along with arsenic removal, it also improves plant productivity under stressful conditions. A crucial aspect of such a strategy is the selection of bacterial inoculum. The described study demonstrates that the indigenous wastewater isolate, ASBT-KP1, could be a promising candidate. Identified as *Klebsiella pneumoniae*, ASBT-KP1 harbors genes associated with heavy metal and oxidative stress resistance, production of antimicrobial compounds and growth-promotion activity. The isolate efficiently accumulated 30 μg/g bacterial dry mass of arsenic. Tolerance toward arsenate and arsenite was 120 mM and 70 mM, respectively. Plant biomass content of *Vigna radiata* improved by 13% when grown in arsenic-free soil under laboratory conditions in the presence of the isolate. The increase became even more significant under the same conditions in the presence of arsenic, recording a 37% increase. The phylogenetic analysis assigned ASBT-KP1 to the clade of *Klebsiella* strains that promote plant growth. Similar results were also observed in *Oryza sativa*, employed to assess the ability of the strain to promote growth, in plants other than *V. radiata.* This study identifies a prospective candidate in ASBT-KP1 that could be employed as a plant growth-promoting rhizoinoculant in agricultural practices.

## 1 Introduction

Soil is a vital component of the terrestrial ecosystem since its influence extends from the growth of plants to the operation of biogeochemical nutrient cycles. Among the pollutants threatening soil fertility, heavy metals are the most severe (Oves et al., 2017). Cadmium, arsenic, mercury, chromium and copper are the most common heavy metal contaminants (Majumder et al., 2013; Hou et al., 2015; Oves et al., 2023). Among them, arsenic is a highly toxic, ubiquitous metalloid released into the environment through natural and man-made activities and is known to cause complications in living beings (Bahar et al., 2013). The presence of arsenic in agricultural soils adversely affects soil biodiversity, fertility, and crop productivity, ultimately resulting in the production of toxic agricultural products due to uptake by edible plant parts leading to bioaccumulation in humans and animals (Deng et al., 2015; Barra Caracciolo and Terenzi, 2021). Chronic exposure to inorganic arsenic disrupts the neurological, respiratory, digestive, endocrine, renal, cardiovascular, and reproductive systems (Mohammed Abdul et al., 2015). Arsenic levels in soil usually range from 1 to 40 mg/kg, but applying pesticides and contaminated water for irrigation further increases this. Studies have found that many countries, including India, the USA, Chile, Bangladesh, and Nepal, have high concentrations of arsenic in their water and soil (Dahal et al., 2008; Murcott, 2012; Wilson, 2015; Raturi et al., 2023). Among the most severely affected areas is the Bengal Delta (West Bengal, Bangladesh, and Nepal), where dissolved arsenic concentrations exceed 200 g/L (Mohanty, 2017). Arsenic is found in four oxidation states in the soil, with the trivalent arsenite, As (III) and pentavalent arsenate, As (V), being the most predominant inorganic forms (Smedley and Kinniburgh, 2002; Bahar et al., 2012). Arsenite being highly soluble, is difficult to remove and hence is more toxic than arsenate (poorly water-soluble, thus reducing its bioavailability) (Neff, 1997; Majumder et al., 2013).

Currently, methods like chemical extraction and excavation, soil washing and flushing, and landfilling are used to remove arsenic from the soil. Due to the health risks associated with arsenic exposure and the high cost of expenditure associated with current remediation techniques, developing cost-effective technologies has become highly imperative. Bioremediation techniques assisted by biomass, microbes (Wang X. et al., 2017), and plants are cost-effective methods attracting widespread attention in removing heavy metal pollutants from agricultural land (Sher et al., 2021). Microbial bioremediation leverages the capability of microorganisms found in wastewater and soil to combat arsenic-induced toxicity by developing various resistance mechanisms that mobilize, immobilize, and transform arsenic into non-toxic forms (Rosen and Tamás, 2010). This resistance mechanism includes arsenic adsorption, accumulation, exclusion, transformation, and precipitation (Mallick et al., 2018). Two communities of arsenic-resistant bacteria help in the microbial remediation of arsenic: (i) those that render protection by uptake and eventual reduction of the bioavailable arsenic (Anderson and Cook, 2004; Cavalca et al., 2010), and (ii) the other that oxidizes arsenite to arsenate (Ghosh et al., 2011; Wang et al., 2011). Arsenic resistance and adaptation in bacteria are acquired through transposons and chromosomal resistance mechanisms or are plasmid-induced. Detoxification of arsenic can be either an inorganic or organoarsenic detoxification process, mediated by arsenic resistance operons. The inorganic process involves *ars* operon-encoded proteins that confer resistance to arsenate, arsenite and arsenicals using the genes a*rsR, arsB,* and *arsC* that encodes proteins arsenite-responsive *trans*-acting transcriptional repressor protein, arsenite antiporter and arsenate reductase, respectively (Hobman and Crossman, 2015). Other than these, *ArsA* and *ArsD* are two genes found in several operons of Gram-negative bacteria that confer arsenic resistance. The organoarsenic detoxification process in bacteria involves *Ars* genes: *ArsL, ArsM* and *ArsH* which encode for the enzymes CeAs bond lyase, As(III) *S*-adenosylmethionine methyltransferase and methylarsenite oxidase, respectively (Yang and Rosen, 2016). One of the techniques commonly used to identify these genes in Gram-positive or Gram-negative bacteria is reverse transcriptase quantitative PCR (Firrincieli et al., 2019). Studies have identified many arsenic-oxidizing and arsenic-accumulating bacteria like *Pseudomonas* spp. (Ghosh et al., 2015), *Klebsiella pneumoniae* (Kumar et al., 2021), *Bacillus* spp. (Dey et al., 2016)*, Acinetobacter junii* (Marwa et al., 2019), and *Pseudomonas alcaligenes* (Zhang et al., 2015) to alleviate arsenic from soil and water alike. These bacteria are usually isolated from soil (Das and Sarkar, 2018), groundwater (Kao et al., 2013; Dey et al., 2016), sediments (Pepi et al., 2007), and root nodules (Seraj et al., 2020). The metabolic diversity of these microbes helps them survive and utilize a variety of substrates in highly complex environments, thus helping their ability to survive and thrive in toxic conditions. Plant-associated bacteria can improve phytoremediation efficiency directly by altering the accumulation of metals in plants and indirectly by promoting plant growth and productivity (Ali et al., 2013). Plants and associated rhizospheric bacteria are a more effective bioremediation strategy for soil and water than plants and bacteria alone (Irshad et al., 2021). For example, the fern species *Pteris vittata* showed enhanced arsenic removal and plant growth promotion when inoculated with a consortium of bacterium, including *Pseudomonas* sp. P1III2, *Pseudoxanthomonas* sp. P4V6, *Bacillus* sp. MPV12, Var*iovorax* sp. P4III4 and *Delftia* sp. P2III5 (Lampis et al., 2015). Thus, arsenic-resistant PGP (Plant Growth Promoting) bacteria are in great demand for arsenic removal from soil and other environments to minimize arsenic accumulation in food crops.

A recent study by Vineethkumar et al. (2020) reported the presence of 20 ppb-43 ppb of arsenic in Kerala’s coastal sediments in the regions of Parayakadavu (9°05′03.9 “N 76°29′14.8” E) and Chavara (8°59′26.8 “N 76°31′23.4” E). Hence, the locale presents a suitable ecosystem for the bioprospection of heavy-metal tolerant bacterial PGPs. Moreover, the successful commercialisation of PGPBs (Plant Growth Promoting Bacteria) requires the development of site-specific, climate-resilient active formulations. Thus, indigenous microorganisms play a vital role in *in situ* bioremediation as it is more straightforward and economical compared to other methods.

The described study explores the potential of ASBT-KP1 in promoting growth in *Vigna radiata* and simultaneously removing arsenic. Plant growth and supportive function, biomass, chlorophyll production and arsenic accumulation in the presence of the isolate in arsenic-spiked soil were studied. The isolate was characterized with regard to plant growth-promoting traits, arsenic tolerance, and pathogenicity. Whole genome sequencing served to identify the isolate and genes associated with PGPR activity, while the 16S rRNA gene sequence was used to establish its phylogenetic relationship with recognized PGP strains. The amenability of this strategy in other plants was tested employing the *Oryza sativa* Kym 2-Bhagya variety, which also expands the prospective application of ASBT-KP1 in established agricultural practices involving different crops.

## 2 Materials and methods

### 2.1 Enrichment and isolation of PGP bacteria from wastewater

Wastewater samples were collected from the Effluent Treatment Plant, Amrita Vishwa Vidyapeetham, Amritapuri campus, Kerala. Ammonium Mineral Salt (AMS) agar supplemented with 20 μM LaCl_3_ and 8% methanol (Skovran and Martinez-Gomez, 2015) was used for isolation and culture. Amphotericin B (1.5 μg/mL) was used as an antifungal agent in the media. ASBT-KP1 is one among 11 priorly isolated bacterial strains from the wastewater collected from the effluent treatment plant (ETP) collection tank of the university campus of Amrita Vishwa Vidyapeetham, Amritapuri, Kerala, India. The collection tank receives sewage and sullage from the septic tanks of different campus departments. ASBT-KP1 was chosen for its maximum arsenic tolerance level when compared to others.

### 2.2 Characterization of ASBT-KP1

The isolate was characterized based on its morphological and physiological characteristics. The bacterial growth curve was studied as described by Anes et al. (2017). The growth optima of the strain ASBT-KP1 was studied at different pH (3–11) and temperatures (4–70°C) after 24 h incubation (Mukherjee et al., 2020). The sugar fermentation profile of the isolate was analyzed using the HiCarbo Kit (KB009A/KB009B1/KB009C) Hi-Media, India, as per the instructions given by the manufacturer. The Kirby-Bauer disk diffusion method was followed to test the antibiotic susceptibility of the isolate (Drew et al., 1972). The following antibiotics were used for the study; Ceftazidime (30 μg), Ceftazidime/clavulanic acid (30/10 μg), Ticarcillin (75 μg), Ciprofloxacin (5 μg), Levofloxacin (5 μg), Co-Trimoxazole (25 μg), Tobramycin (10 μg), Aztreonam (30 μg), Meropenem (10 μg), Colistin (10 μg), Imipenem (10 μg), Gentamicin (120 μg), Piperacillin (30 μg), Minocycline (30 μg), Amikacin (30 μg).

### 2.3 Whole genome sequencing of ASBT-KP1

The genomic DNA (gDNA) of ASBT-KP1 was isolated using the protocol described in Sambrook and Russel, Fourth edition. The isolated DNA was used for whole genome shotgun sequencing and analysis using the Illumina HiSeq 2,500 Sequencing platform at DNAxperts Pvt. Ltd. (India). The gDNA sample was used to construct pair-end sequencing libraries using the Illumina Truseq protocol v3. The average fragment sizes for the pair-end libraries were 2 × 100/2 × 150 bp. The *de novo* assembly and annotation of the gDNA sample were carried out using the tools from PATRIC (Wattam et al., 2017, 2018) as per the user’s manual. The strain identification and annotation were carried out using RASTtk. Moreover, the predicted and annotated genes were compared manually with *Klebsiella* sp. D5A genome data (Liu et al., 2016), a known PGP bacteria for the presence of genes responsible for the PGP traits.

### 2.4 Phylogenetic analysis

The BLASTN analysis^1^ for identifying the closely related bacterial species was performed with the 16S rDNA sequence of ASBT-KP1 (Accession number – MT815532.1). The known and closely related bacterial species of ASBT-KP1 and known PGP *K. pneumoniae* strains were first aligned using ClustalW with the Gap opening penalty and Gap extension penalty for Pairwise and multiple alignments set at 10.00 and 1.00, respectively. The aligned sequence was then used for phylogenetic tree construction using the Neighbour-Joining method (Saitou and Nei, 1987) with the Partial deletion value set at 80%. The evolutionary distances were computed using the maximum composite likelihood method (bootstrap values, as a percentage of 1,000 replicates) (Tamura et al., 2004) using the MEGA11 software (Tamura et al., 2021). *Erwinia persicina* strain LMG 11254 was used as the outgroup.

### 2.5 Quantitative estimation of PGP traits of ASBT-KP1

Plant Growth Promoting traits, including Indole Acetic Acid (IAA) production, siderophore production, precursor 1-aminocyclopropane-1-carboxylate (ACC) deaminase enzyme production, phosphate solubilization, Hydrocyanic acid (HCN) production and production of extracellular ammonia for the strain ASBT-KP1 were analyzed. The ability of ASBT-KP1 to produce IAA was detected by the Salkowski method (Glickmann and Dessaux, 1995; Gang et al., 2019). The siderophore production was tested using a modified blue agar chrome azurol sulfonate (CAS) assay protocol by Schwyn and Neilands (Louden et al., 2011; Arora and Verma, 2017). Production of an orange-yellow zone around the inoculated bacterial colonies indicated a positive reaction. For measuring the ACC deaminase activity, the method described by the researchers Penrose and Glick was followed (Penrose and Glick, 2003; Mukherjee et al., 2020). Bradford method was used for estimating the protein concentration of the samples (Kruger, 2009). The phosphate solubilization ability of ASBT-KP1 was tested on Pikovskaya agar medium (M520, HiMedia, India) plates. The presence of a halo zone around the colony indicated a positive result. Phosphate solubilization was estimated using the modified phospho-molybdenum method described by the researchers (Wang Z. et al., 2017). The resultant blue color was measured at 700 nm. The ability of ASBT-KP1 to produce ammonia was tested in peptone water following the protocol of Ahmad et al. (2008). A change in color from yellow to brown with the precipitate formation on adding Nessler’s reagent indicated a positive result. The HCN production was studied as per the protocol (Indiragandhi et al., 2008). A color change from yellow to reddish brown indicated HCN production.

### 2.6 Salinity and heavy metal tolerance

The salt tolerance for the isolate was studied by culturing it in R2-A broth supplemented with NaCl at different concentrations – 0, 0.1, 0.5, 1, 2, 5, 10, and 20% for 24 h at 37 ± 2°C. Absorbance at 600 nm was measured following incubation. Heavy metal tolerance of the isolate was studied in R2-A broth supplemented with various concentrations (1 μM–10 mM) of heavy metals. The heavy metals used were copper (II) (CuSO_4_·5H_2_O), arsenate (V) (Na_2_HAsO_4_. 7H_2_O), arsenite (III) (NaHAsO_4_) and mercury (II) (HgCl_2_). Growth of ASBT-KP1 was estimated by serially diluting and plating the culture suspension onto LB plates to determine the colony count after incubation for 48 h at 37°C (Mukherjee et al., 2020). The resistance exhibited by ASBT-KP1 to Arsenite (III) and Arsenate (V) was studied in R2-A broth supplemented with increasing concentrations of As (III), from 0 to 100 mM and As (V), from 0 to 150 mM. The negative control for the experiment was the bacterial cultures without As (III) and As (V) treatment. The treated and untreated cells were incubated at 37°C for 48 h. The growth was measured at OD_600nm_ in a microplate reader (BioTek, United States) and serially diluted and plated onto LB plates to determine the colony count. The minimal inhibitory concentrations (MIC) of As (III) and As (V) were determined for ASBT-KP1.

### 2.7 Analyzing the effect of arsenic on ASBT-KP1 using SEM–EDX and ICP-MS

Scanning electron micrographic images were used to analyze the morphological changes in the bacterial population in the presence of 1 mM of arsenate. Untreated bacterial cells were kept as a control. The samples for SEM were prepared as described by Chakravarty and Banerjee (2008). Following incubation for 48 h, the treated and control cells were harvested (7,000 × *g,* 5 min, 4°C) and washed 3 times with 0.1 M phosphate-buffered saline (PBS, pH 7.4). The cells were then fixed overnight with 2% glutaraldehyde at 4°C. The cells were again washed thrice with 0.1 M PBS and then dehydrated with alcohol concentrations of 10, 30, 50, 70, and 90% and absolute ethanol. Before imaging, the samples were placed on glass slides (1 cm × 1 cm) and sputter-coated with gold. The Jeol 6390LAv was used for the SEM imaging (Focardi et al., 2010; Mallick et al., 2018). An energy dispersive X-ray spectrometry (EDX) was carried out with an Oxford XMX N (Oxford Instruments, United Kingdom) probe to estimate the adsorption of As onto the cells.

The arsenic bioaccumulation capability of ASBT-KP1 was determined by ICP-MS analysis of the bacterial sample grown in 25 mL of R2-A broth supplemented with 1 mM of As (V) for 72 h. ASBT-KP1 culture was pelleted down and washed with sterile water, followed by a wash with 0.1 M of EDTA to remove any arsenic adsorbed to the surface of the cells. The sample was washed twice with sterile water and dried overnight at 70°C. The sample was weighed and digested with concentrated nitric acid before being analyzed in an iCAP RQ (Thermo Scientific) using Helium KED mode.

### 2.8 Biofilm formation

The isolate was studied for its ability to form biofilm using the quantitative biofilm assay described by the researchers (Gómez et al., 2016). On a sterile 24-well plate (Tarson, Korea), R2-A broth supplemented with arsenic (50 mM) and bacterial suspension of ASBT-KP1 of OD_600nm_ 0.5 ± 0.05 was added to each well with four replicates and incubated overnight at 37°C. The plates were washed thrice with 2 mL of sterile distilled water, after which 2 mL of methanol was added to each well to fix the biofilm for 15 min. After incubation, methanol was removed, and 2% (v/v) of crystal violet was added and stained for 5 min. The plate was placed under gently running tap water to remove excess stains. The stain from the adherent cells was released by adding 2 mL of 33% (v/v) glacial acetic acid. The absorbance in each well was measured at 595 nm in a microplate reader (BioTek, United States). R2-A broth devoid of culture was kept as the negative control. The negative control’s mean OD_600nm_ value was kept as the OD cut-off value (ODC). Based on the OD_600nm_, the strain was classified as strong biofilm producers if their OD > ODC × 4. The strains are said to be moderate biofilm producers if 2 × ODC < OD ≤ 4 × ODC, weak biofilm producers if ODC < OD ≤ 2 × ODC, and non-biofilm producers if OD ≤ ODC (Borges et al., 2012).

### 2.9 *In vitro* study of *Vigna radiata* under arsenic stress

To study the plant growth promotion effect of ASBT-KP1 on *V. radiata*, the seeds of *V. radiata* were procured from the local market in bulk to keep the sample uniform for all the experiments. The seeds and the resulting plants were identified as *V. radiata* and assigned an accession number SNCH 4518 by Dr. Kiranraj MS, Curator of Herbarium, Sree Narayana College Herbarium, Sree Narayana College, Kollam, Kerala. The study was conducted according to the local and national guidelines for the cultivation of plants. Surface sterilized seeds of *V. radiata* were placed in Petri plates with Murashige and Skoog medium (PT103, HiMedia) for germination at 25–27°C for 3 days in the dark. The seedlings were bacterized with ASBT-KP1 for 8 h and then planted in seedling trays. The soil for the study was collected from a previously wastewater-irrigated site in Kollam, Kerala, India.

The basic properties of the soil are as follows: pH, 5.5; total N, 0.14%; total organic carbon, 0.16%; available phosphate, 16.7 mg/kg; total soil As content was below the detection limit of 0.1 mg/kg (Supplementary Table S3). The soil was sieved and sterilized before being used for the study. To study the effect of arsenic toxicity, seedlings were grown in seedling trays filled with soil supplemented with 10 mg/kg of sodium arsenate, which was relevant to the As concentrations recorded in polluted soil environments (Majumder et al., 2013). The soil was left for proper acclimatization of arsenic for 1 week before use. The planted seedlings were rhizoinoculated with ASBT-KP1 (10^8^ CFU/mL); in the soil, the bacterial load was 10^7^ CFU/g. Soil, without culture on the seedlings, was kept as control. The plants were harvested after 21 days, and their morphological and biochemical growth parameters were analyzed. The morphological parameters studied were shoot length, root length and dry biomass. Biochemical parameter, chlorophyll (Chl-a. chl-b, and total chl.) content was measured. The arsenic uptake by *V. radiata* from the soil was determined by ICP-MS analysis of the digested plant samples.

The rhizospheric soil from ASBT-KP1 treated plants and untreated control was collected to study the ability of ASBT-KP1 to establish itself in the rhizospheric soil. The soil was serially diluted and cultured on nutrient-rich media, LB and *Klebsiella* spp. specific media, *Klebsiella* Blue agar (KBA) (Prasad et al., 2022). The systemic uptake of ASBT-KP1 was tested by inoculating 7-day-old *V. radiata* seedlings grown in unsterile soil with 2 mL of ASBT-KP1 (10^8^ CFU/mL). Uninoculated plants were kept as control. Six plants were randomly selected from each group and surface sterilized with 2% sodium hypochlorite supplemented with 0.1% Tween 20, followed by three washes with sterile distilled water (Sahu et al., 2022). The samples were macerated and plated onto LB and KBA media and incubated at 37°C for 24 h. Growth in KBA media indicates the presence of ASBT-KP1, owing to the specificity of the media.

### 2.10 *In vitro* study of *Oryza sativa* under arsenic stress

Seven-day-old *O. sativa* Kym 2 – Bhagya variety (seeds procured from Onattukara Regional Agricultural Research Station, Kerala Agricultural University, Kayamkulam, Kerala, India) saplings were replanted onto non-sterile soil spiked with 10 mg/kg of sodium arsenate. Three sets of 12 saplings each were employed for test and control experiments. Rhizoinoculation of ASBT-KP1 was performed as described in section 2.11, involving the *V. radiata* experiment. The plants were harvested after 15 days, and their morphological and biochemical growth parameters were analyzed. The morphological parameters studied were shoot length, root length and dry biomass. The arsenic uptake by *O. sativa* from the soil was determined by ICP-MS analysis of the digested plant samples.

The ability of ASBT-KP1 to establish itself in the rhizospheric soil of *O. sativa* was studied as previously described in section 2.11, for *V. radiata*. The systemic uptake of ASBT-KP1 was tested by plating the surface sterilized parts of plants onto KBA media. Growth in KBA media indicates the presence of ASBT-KP1, owing to the specificity of the media.

### 2.11 *Caenorhabditis elegans*-based virulence testing

ASBT-KP1 was assessed for its virulence using two different methods. Firstly, the string test was performed to check for hypervirulence, as described by the researchers (Hagiya et al., 2014). Secondly, *Caenorhabditis elegans* was used for studying the virulence of ASBT-KP1 (Salim et al., 2022). *Caenorhabditis elegans* strain N2 (wildtype strain) were grown in nematode growth media (NGM; Peptone 2.5 g/L, NaCl 2.9 g/L, Bacto-Agar 17 g/L, CaCl_2_ 1 mM, cholesterol 5 μg/mL, KH_2_PO_4_ 25 mM, MgSO_4_ 1 mM) having a lawn culture of *E. coli* OP50. The *C. elegans* larvae were synchronized by isolating the eggs from gravid adults using a sucrose density-gradient-based separation technique (Shaham, 2006). The isolated eggs were hatched overnight in M9 buffer supplemented with cholesterol (1 mg/mL). The L1-stage worms were resuspended in M9 buffer, and the test bacteria –ASBT-KP1 (washed with sterile distilled water and resuspended in M9 buffer at an OD of 0.1). Uninfected *C. elegans* with *E. coli* OP50 was used as a control. The survival of the L1 worms to adults for the next 5 days was observed and plotted as a survival curve using Kaplan and Meier survival plot using Graph Pad Prism (v9.0.0).

### 2.12 Statistical analysis

The data collected were analyzed statistically using Graph Pad Prism (V 9.0). All experiments were conducted in triplicates or otherwise specified. The statistical difference for the plant growth studies was assessed using the One-way ANOVA followed by Dunnett’s multiple comparisons test for multiple comparisons between the control and treated samples. The *O. sativa* growth studies were tested using Welch’s *t*-test to compare control and test samples. The data were represented as the mean ± standard deviation of replicates, and the significance level was studied at a *p*-value <0.05.

## 3 Results

### 3.1 Identification and characterization of ASBT-KP1

**Figure 1:**
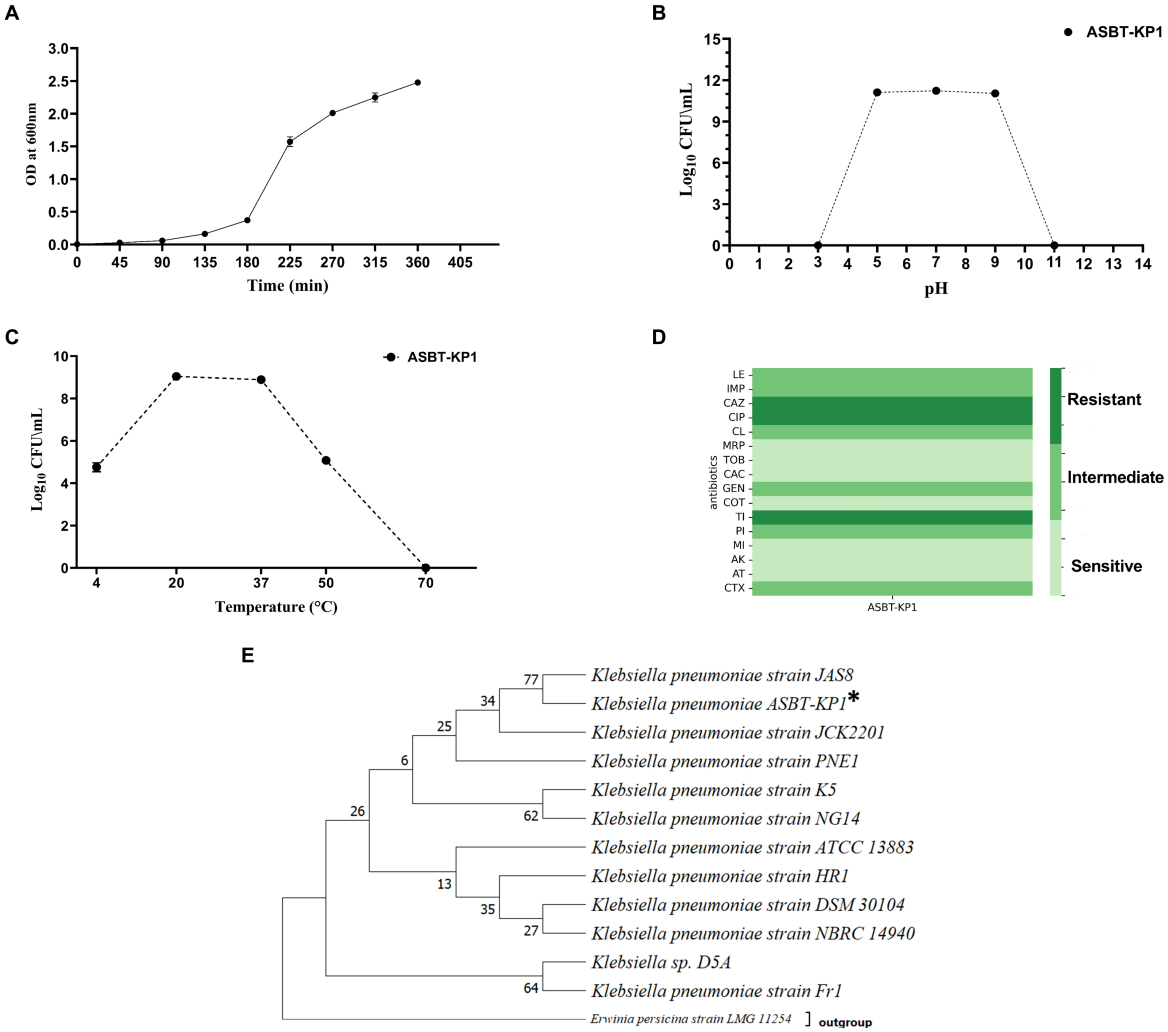
Growth characteristics, antibiogram and phylogenetic tree of ASBT-KP1. **(A)** Growth curve to estimate the generation time; 29 min. **(B)** Optimal pH and **(C)** temperature of ASBT-KP1. **(D)** Antibiotic susceptibility profile (LE, levofloxacin; IMP, imipenem; CAZ., ceftazidime; CIP, ciprofloxacin; CL, colistin; MRP, meropenem; TOB, tobramycin; CAC, ceftazidime/clavulanic acid; GEN, gentamicin; COT, co-trimoxazole; TI, ticarcillin; PI, piperacillin; MI, minocycline; AK, amikacin; AT, aztreonam and, CTX, cefotaxime). **(E)** Phylogenetic tree demonstrating the evolutionary relationship of the strain with other plant growth promoting *Klebsiella* spp., using MEGA 11. ‘*’ shows the studied strain, ASBT-KP1.

ASBT-KP1 is a non-motile bacillus of a size range of 1.2–2.3 μm long. The sugar utilization profile of ASBT-KP1 includes lactose, xylose, fructose, dextrose, trehalose, sucrose, l-arabinose, mannose, galactose, inositol, maltose, adonitol, and arabitol but not d-arabinose, sorbose, inulin, glycerol, and dulcitol (Supplementary Table S1). The generation time of the isolate is 29 min (Figure 1A). The organism grew in a pH and temperature range of 5–9 pH (Figure 1B) and 4–50°C, respectively (Figure 1C). ASBT-KP1 showed sensitivity to all the tested antibiotics except ceftazidime, ciprofloxacin and ticarcillin (Figure 1D). Based on the whole genome sequence analysis, ASBT-KP1 is identified as *K. pneumoniae*. Throughout the manuscript, ASBT-KP1 is used as the strain designation, implying that it is *Klebsiella pneumoniae.* The phylogenetic tree of ASBT-KP1 was constructed using 16S rRNA gene sequences of known and closely related bacterial species and those of PGP *K. pneumoniae* strains (Figure 1E) (Tamura et al., 2021). The evolutionary relatedness of ASBT-KP1 reiterates its potential as a PGPB.

### 3.2 ASBT-KP1 genome

**Figure 2:**
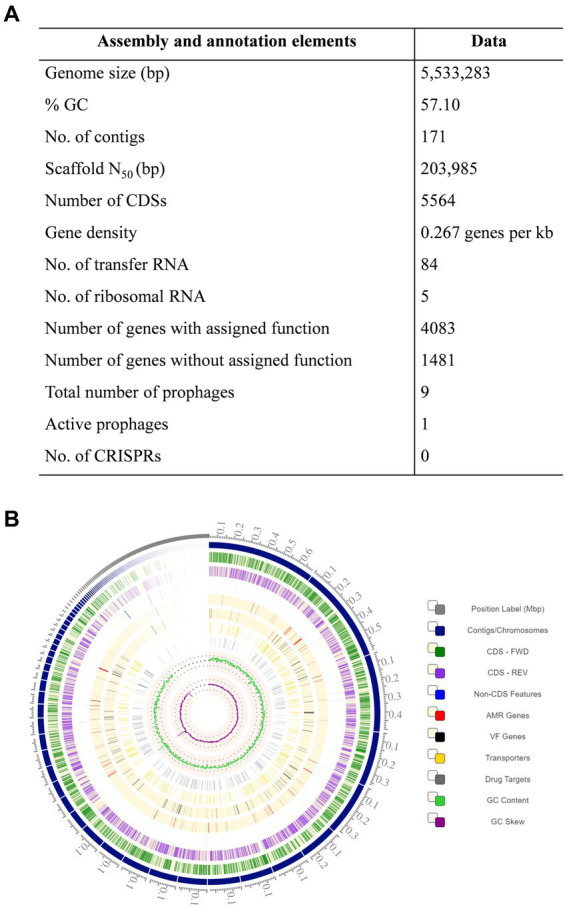
Whole genome analysis of ASBT-KP1. **(A)** Genomic features of *K. pneumoniae* strain ASBT-KP1 and **(B)** the circular genome of *K. pneumoniae* strain ASBT-KP1 with CDS in the forward (Dark green) and the reverse (Violet) strand constructed using Circos in BV-BRC (https://www.bv-brc.org/).

The genome of *K. pneumoniae* ASBT-KP1 has a total of 5,533,283 bp with an average G + C content of 57.10% (Figure 2A). The genome has 5,564 coding sequences, of which 4,775 genes of the predicted CDS were assigned biological roles while 789 coding sequences were classified as proteins with hypothetical functions. ASBT-KP1 has five rRNA genes comprising two 5S rRNA, two 16S rRNA, and a 23S rRNA. Moreover, it has 84 tRNA genes representing 21 amino acids and one pseudo tRNA.

### 3.3 Genotypic PGP characterization

Supplementary Table S2 summarizes the genes in the ASBT-KP1 attributed to phosphate solubilization and uptake, Indole Acetic Acid production, siderophore synthesis, resistance to oxidative stress, and resistance to heavy metals like arsenic, copper, zinc, and cadmium. Phosphate solubilization genes include those that encode glucose dehydrogenase (GDH) and redox cofactor Pyrrolo-quinolone quinine (pqq) and *pqqBDEF,* responsible for the synthesis of gluconic acid and *PstBACS* and *PhnCDE2E1.*The strain encodes 8 copies of genes for siderophore receptors and genes responsible for the synthesis of siderophores *viz.*, TonB-dependent receptors, IroN receptors, ferrichrome-iron receptor, TonB-dependent hemin ferrichrome receptor, TonB-dependent ferric enterobactin, colicins B, D receptors, *entBF* and *entS*. Additionally, the strain has 49 genes that encode for iron transport proteins. The presence of ABC transporter and associated genes indicate that the strain can heterologously obtain siderophores produced by other soil bacteria. Genes that confer resistance to heavy metals like arsenic (a*rsCBRD*), copper (*copDC* and related genes), and zinc are present in the strain. The genome has an *ars* gene cluster that confers arsenic resistance through the production of arsenate reductase. From the circos plot (Figure 2B), it could be deduced that the genes for arsenical resistance operon repressor (*arsR*), arsenite permease (*arsB*) and arsenate reductase (*arsC*) exhibit strong synteny (contig 4, fig|1162296.25.peg.1694–1,696). These genes code for a transcription repressor, an expulsion pump, and a reductase enzyme essential in the arsenic bioremediation potential of ASBT-KP1. Genes that protect plants against phytopathogens are hosted by ASBT-KP1, such as *phzF,* involved in phenazine synthesis, which functions as an antibiotic. A gene which encodes chitinase enzyme, known to degrade the cell walls of pathogenic fungal and insect pests, is also present.

Additionally, the strain has *gabR* and associated genes involved in gamma-aminobutyric acid production. The genome also encoded proteins that protect the cell from oxidative stress: three superoxide dismutases, five glutathione-*S*-transferases, and six peroxidases. The antibiotic resistance profile of ASBT-KP1 indicates its sensitivity to most tested antibiotic classes (Figure 1E). This observation was confirmed by an *in vitro* antibiotic susceptibility test followed by validation using the ResFinder tool hosted at CGE (Supplementary Data Sheet 1). Additionally, none of the classic antibiotic-resistance point mutations indicative of functional *ramR, acrR, ompK36, ompK35, ompK37, gyrA, gyrB, rpsL* or *parC* genes are identified that accounts for the sensitivity profile of ASBT-KP1.

### 3.4 Phenotypic PGP characterization

ASBT-KP1 produced 120 ± 5 μg/mL of IAA (Table 1). The strain showed an increase in the production of IAA in the presence of arsenite (III), 165 μg/mL, and arsenate (V), 270 ± 5 μg/mL. The strain solubilized the calcium phosphate in the medium with a distinct zone of clearance (Supplementary Figure S1A). Qualitatively, 210 ± 5 mM of phosphate was solubilized. ASBT-KP1 expressed ACC deaminase with a specific 4.04 nmol/mg/h activity. HCN production was observed with a color change in the media from yellow to darker yellow-orange (Supplementary Figure S1B).

**Table 1:** Plant growth-promoting traits of the isolate ASBT-KP1.

Plant growth-promoting traits	Data
Ammonia production (μM)	1,690 ± 5
IAA concentration of (μg/mL)	120 ± 5
IAA concentration (μg/mL) in presence of As (V)	270 ± 5
IAA concentration (μg/mL) in presence of As (III)	165 ± 5
Concentration of soluble phosphate (mM)	210 ± 5
ACC deaminase activity (nmol g^−1^ h^−1^)	4.04
HCN production	Positive

### 3.5 Heavy metal and salinity tolerance

**Figure 3:**
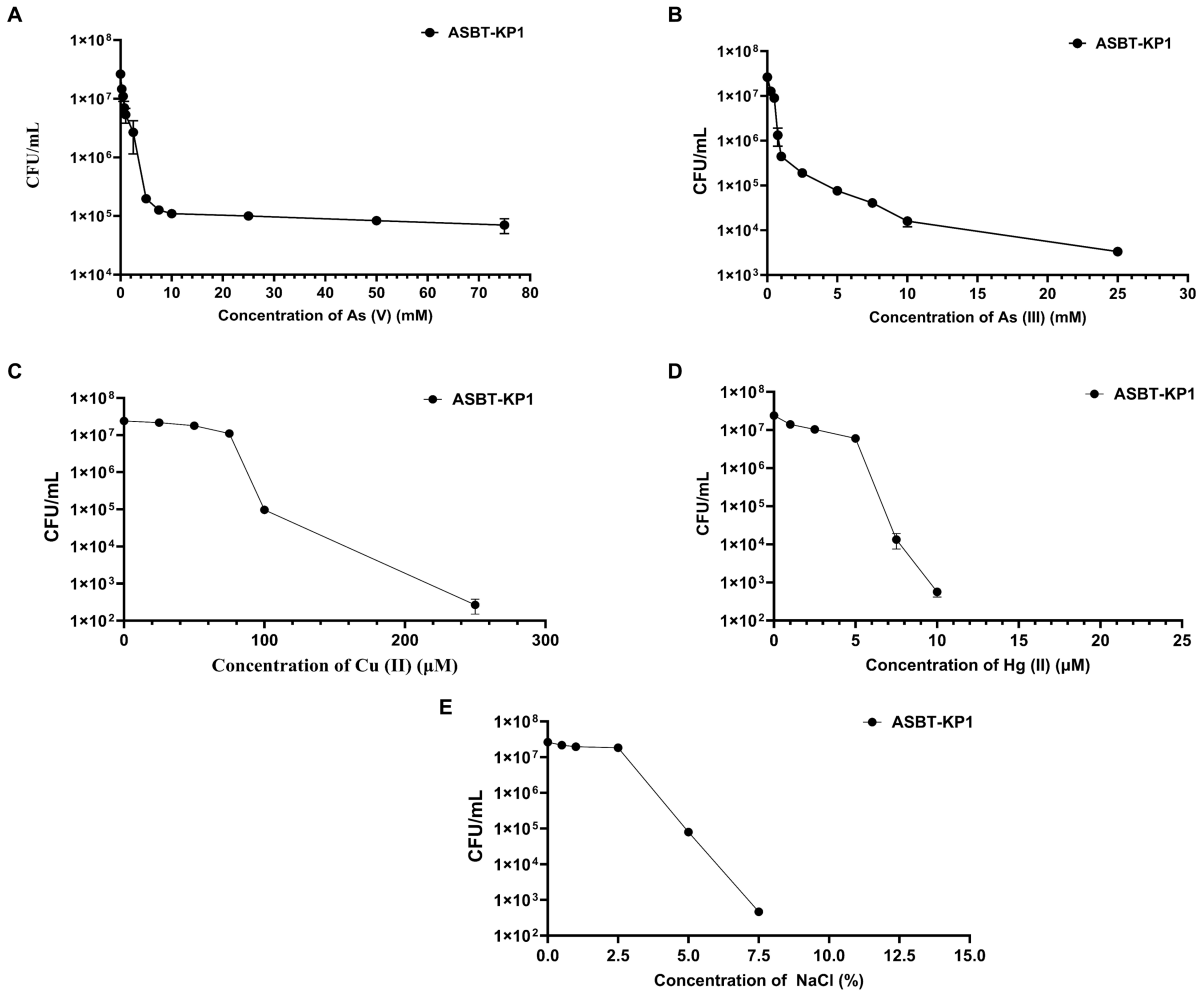
Heavy metal tolerance and halotolerance of ASBT-KP1. Tolerance toward **(A)** As (V); **(B)** As (III); **(C)** Cu (II); **(D)** Hg (II); and **(E)** NaCl.

The strain showed differential tolerance levels toward mercury, arsenate, arsenite and copper at different concentrations. ASBT-KP1 tolerated >75 mM of As (V) (Figure 3A), >25 mM of As (III) (Figure 3B), ~250 μM of Cu (II) (Figure 3C), and ~ 10 μM of HgCl_2_ (Figure 3D). ASBT-KP1 showed a tolerance of up to 7.5% of NaCl (Figure 3E).

**Figure 4:**
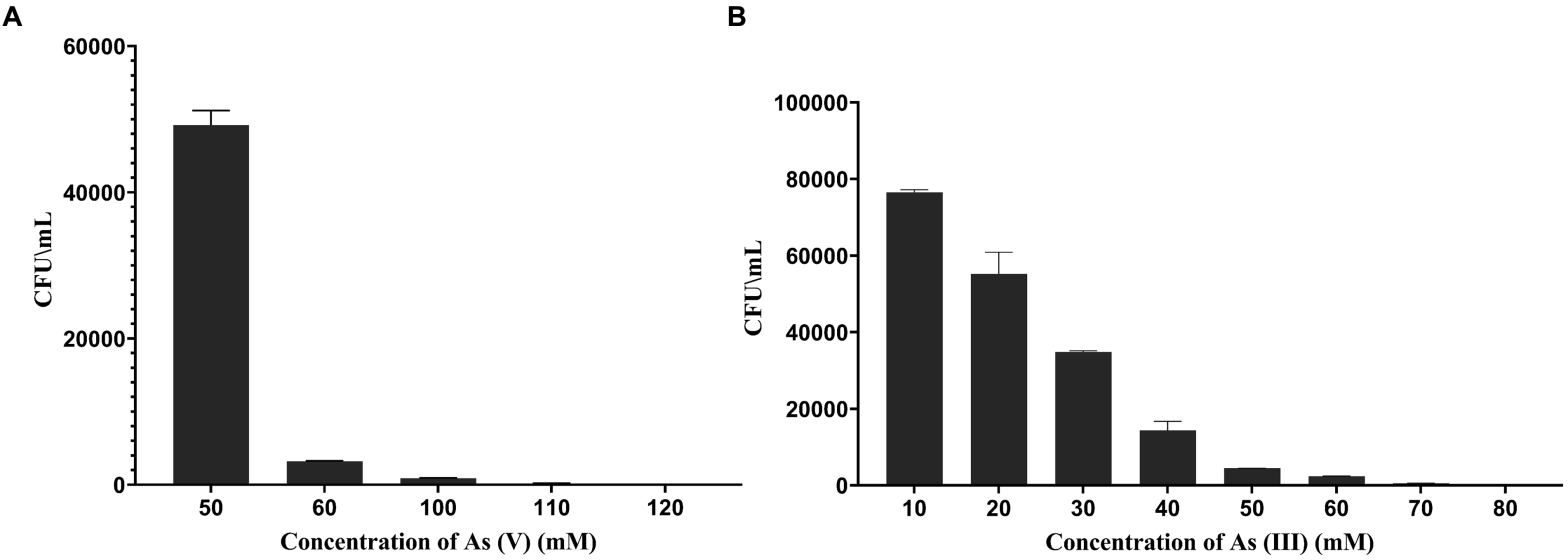
Minimum inhibitory concentration of ASBT-KP1 toward different forms of arsenic. The tolerance of ASBT-KP1 toward **(A)** As (V) and **(B)** As (III).

The minimum inhibitory concentration for the strain against arsenate (V) and arsenite (III) was 120 and 70 mM, respectively. ASBT-KP1 has a relatively reduced tolerance to arsenite (III) compared to arsenate (V) (Figures 4A,B).

### 3.6 SEM, SEM–EDX and ICP-MS analysis of arsenate-treated ASBT-KP1

**Figure 5:**
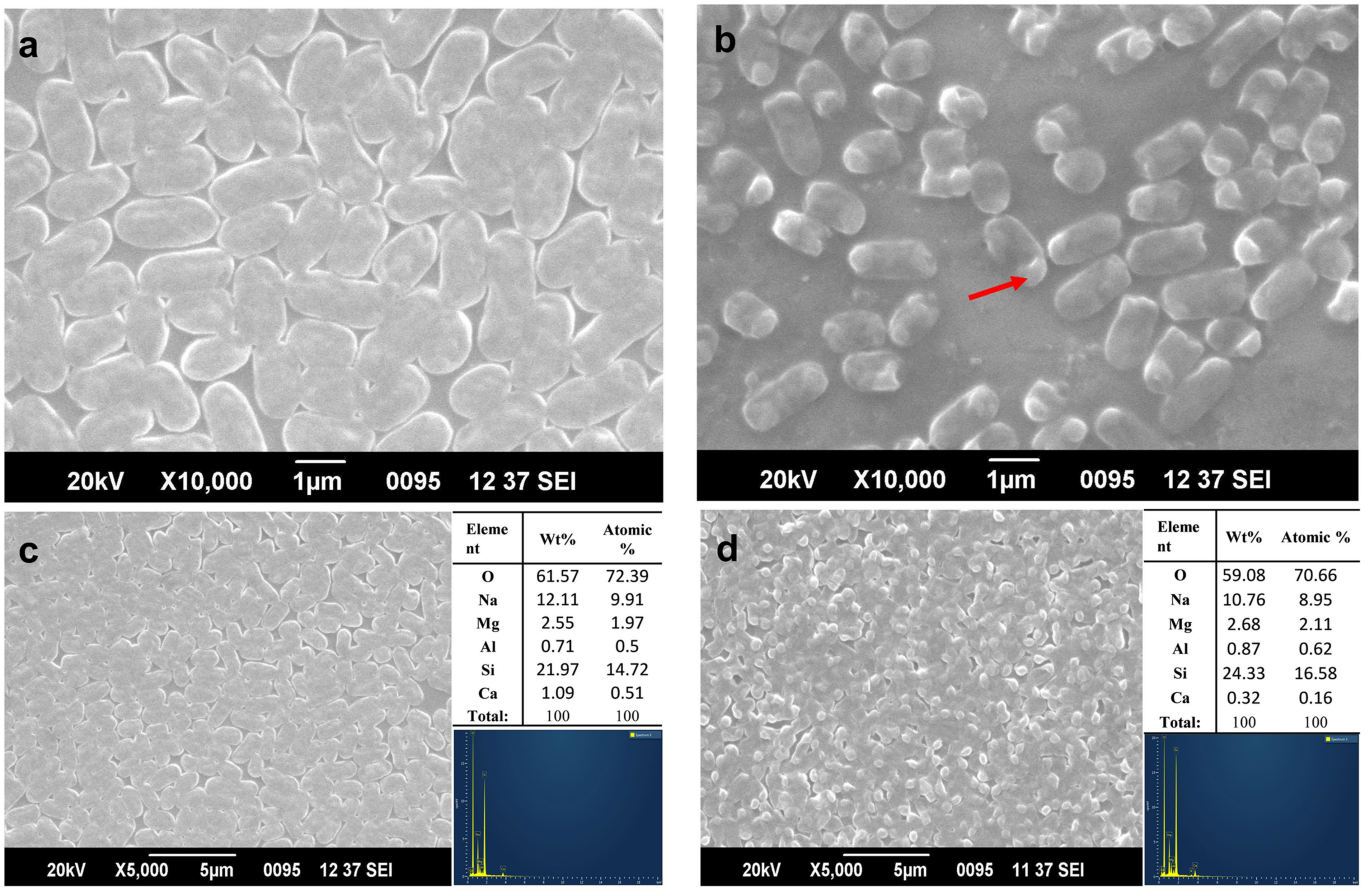
Morphological changes in ASBT-KP1 **(A)** without As (V), and **(B)** with 1 mM of As (V). The red arrow in **(B)** shows the vacuole formation in the treated cells. SEM-EDX analysis of ASBT-KP1 **(C)** without As (V) and **(D)** with 1 mM of As (V), respectively.

Scanning electron micrograph revealed that the arsenic-treated cells showed changes in the morphology and size of the cells. The average size of untreated cells was 1.7 ± 0.5 μm (Figure 5A), while the treated cells shrunk in size with an average decrease of 0.79 ± 0.25 μm (Figure 5B). The outer membrane of the cells did not show any significant changes, while there was visible vacuolation due to As (V) stress (Figure 5B).

The surface of the treated and control cells was further analyzed using SEM–EDX to study the arsenic adsorption by the bacterial cells. The surface of neither treated nor control cells showed any peak corresponding to arsenic (Figures 5C,D), indicating the absence of arsenic adsorption onto the cell surface. The buffer solution, glass slide and coating material contributed to the few unrelated peaks obtained in the analysis. The bioaccumulation potential was determined using ICP-MS analysis of the cell pellet. The analysis showed that the strain accumulated 30 ± 2 μg/g of bacterial dry mass when incubated in 1 mM of arsenate (V) for 72 h.

### 3.7 Biofilm production

The microtiter plate assay for biofilm formation showed that ASBT-KP1 was a strong biofilm producer with an OD_600nm_ of 1.21, 14 times greater than the OD cut-off value (Borges et al., 2012). The isolate also formed biofilm in the presence of arsenate (Supplementary Figure S2).

### 3.8 Effect of ASBT-KP1 on *Vigna radiata*

**Figure 6:**
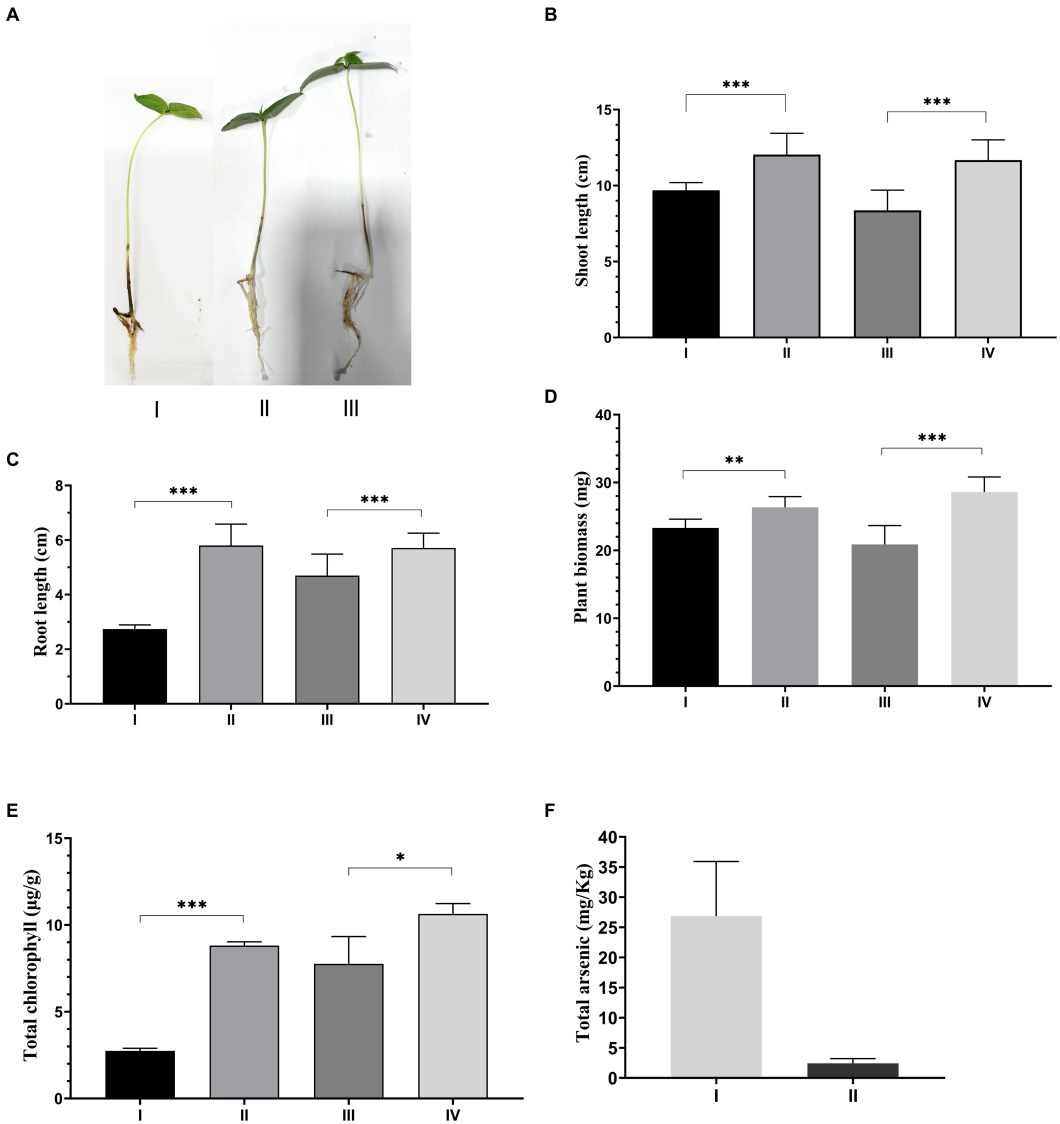
Effect of ASBT-KP1 on arsenic stress induced *V. radiata*. **(A)** Phenotypic expression of the *V. radiata* plants under arsenic stress: plant (I), plant under As stress (II), and plant under As stress treated with ASBT-KP1(III). Estimation of different plant growth-promoting properties of ASBT-KP1 under As stress **(B)** shoot length; **(C)** root length; **(D)** plant biomass; **(E)** total chlorophyll content [I – only plant, II – plant + ASBT-KP1, III – plant + As, IV – plant + As + ASBT-KP1] and; **(F)** As accumulation in the *V. radiata* treated with ASBT-KP1 compared to control sets without any inoculation [I = plant +As, II = plant + ASBT-KP1 + As]. The symbols “*, **, ***” indicate the statistical significance with, * – *p* ≤ 0.05, ** – *p* ≤ 0.01 and *** – *p* ≤ 0.001.

The pH of the soil, total suspended solids, organic carbon, nitrogen, total phosphate, and arsenic levels were measured (Supplementary Table S3) to characterize the soil used for the study. The soil pH was slightly acidic (pH 5.5), and the total organic carbon was 0.78%. The plant growth study revealed that the mean dry plant weight (26.34 ± 0.402 mg) of plants inoculated with ASBT-KP1 (Figure 6D) was significantly higher than (*p* < 0.001) than the uninoculated plants (23.31 ± 0.257 mg). Similarly, the chlorophyll content of the plants inoculated with ASBT-KP1 (8.81 ± 0.125 μg/g) was significantly increased than the uninoculated plants (2.74 ± 0.089 μg/g) (Figure 6E). Statistical analysis of growth, in terms of mean shoot and root length, revealed that the inoculated *V. radiata* plants exhibited a more significant growth (9.6 ± 0.506 cm) when compared to the uninoculated plants (12.0 ± 1.40 cm) (*p* < 0.001). In the presence of Arsenic, *V. radiata* showed an increased mean shoot length (11.7 ± 1.32 cm) mean dry biomass (28.6 ± 2.24 mg) and chlorophyll content (10.6 ± 0.59 μg/g) compared to those without bacterial inoculation (8.37 ± 1.33 cm; 20.9 ± 2.77 mg; 7.77 ± 1.57 μg/g respectively) (Figures 6B–E), (*p* < 0.001). It is evident from the results that the strain protects the plants from the toxic effects of arsenic and improves growth compared to the uninoculated control. Even under normal physicochemical conditions, i.e., in the arsenic-free condition, ASBT-KP1 inoculated plants showed significant improvement in growth. The colonization of the ASBT-KP1 in the rhizospheric environment of the *V. radiata* plants helped significantly decrease the plants’ arsenic uptake by 91% (Figure 6F).

The bacterial load in the test soil was higher (5.5 × 10^6^ CFU/mL) in comparison to that in the control soil (3.5 × 10^6^ CFU/mL) when quantified in nutrient-rich media. ASBT-KP1 was effectively established after its introduction since its presence was detected (1.4 × 10^5^ CFU/mL) after 8 days of incubation. Additionally, to test the infiltration of ASBT-KP1 into the various parts, the plant’s root, stem and leaves were tested for its presence in both LB and KBA. Except in roots (8.2 × 10^3^ CFU/mL), none of the parts showed the presence of the strain. However, other bacterial endophytes were uniformly present in both the control (root – 1.93 × 10^5^ CFU/mL; stem – 1.33 × 10^5^ CFU/mL; leaves – 8.67 × 10^4^ CFU/mL) and test (root – 2.4 × 10^5^ CFU/mL; stem – 1.4 × 10^5^ CFU/mL; leaves – 8.67 × 10^4^ CFU/mL).

### 3.9 Effect of ASBT-KP1 in *O. sativa*

The study on *O. sativa* (Supplementary Figure S3) revealed that the mean dry plant weight (42.8 ± 6.31 mg) of plants inoculated with ASBT-KP1 in the presence of arsenate was higher (Supplementary Figure S4D) than the uninoculated plants (37.6 ± 6.06 mg). The analysis of growth in terms of mean shoot and root length, revealed that the inoculated *O. sativa* exhibited a significantly higher shoot and root length (19.9 ± 2.51 cm; 6.83 ± 1.46 cm) (Supplementary Figures S4A,B) when compared to uninoculated plants (17 ± 2.08 cm; 5.38 ± 1.28 cm) (*p* < 0.05). Thus, from the results, we can ascertain the ability of ASBT-KP1 to improve the growth of the *O. sativa* plants under arsenic-stressed conditions. By colonizing the roots of *O. sativa*, ASBT-KP1 significantly reduced the plants’ arsenic uptake by 74.19% (Supplementary Figure S4E).

The soil inoculated with ASBT-KP1 showed a higher total bacterial load (1.11 × 10^6^ CFU/mL) than untreated soil (9.5 × 10^5^ CFU/mL) when cultured in a nutrient-rich media. The test soil had 5.9 × 10^4^ CFU/mL of ASBT-KP1 indicating its ability to establish itself in the rhizosphere of the *O. sativa* plants.

### 3.10 Survival of ASBT-KP1 infected *C. elegans*

**Figure 7:**
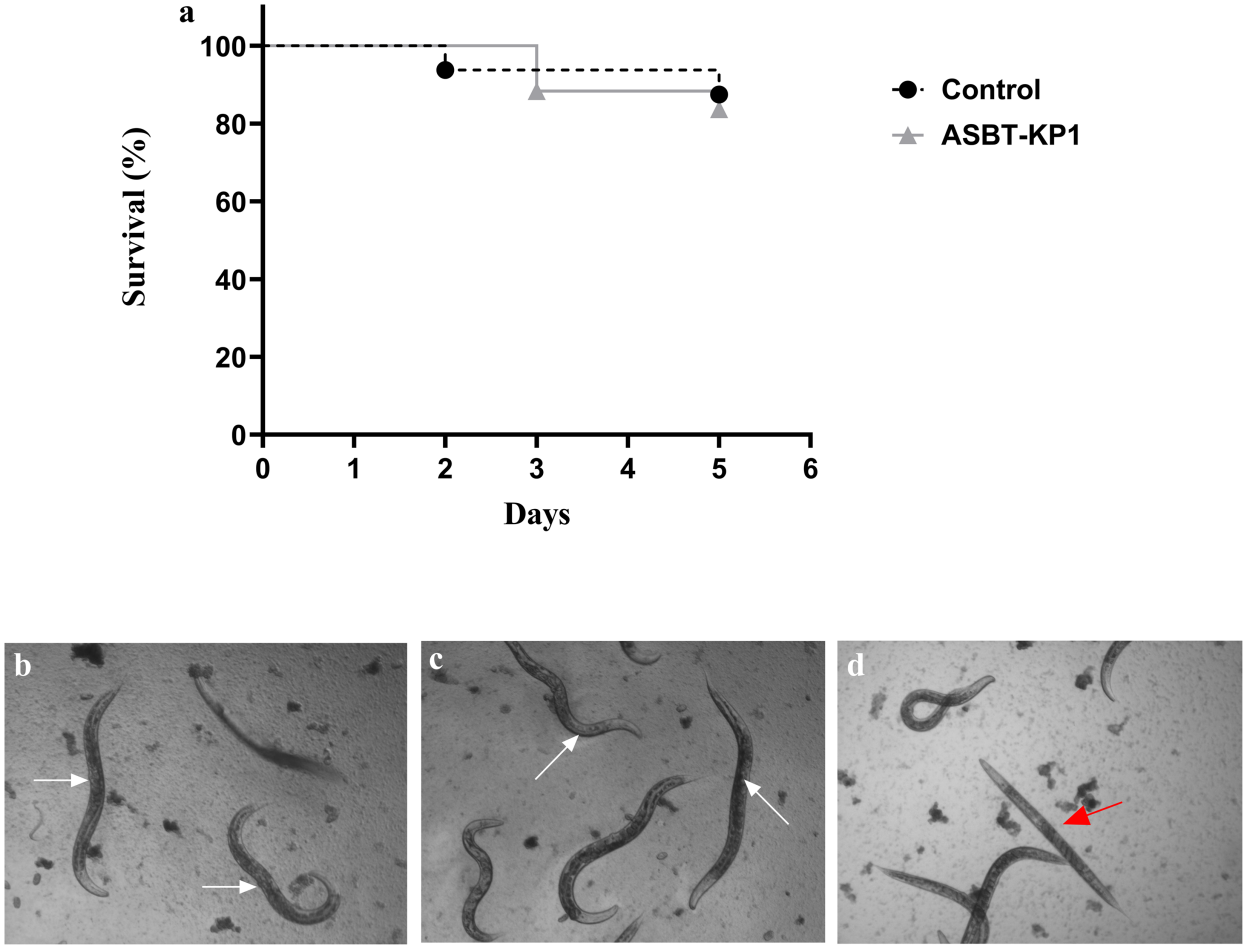
Survival assay of *C. elegans* after treatment with ASBT-KP1. **(A)** Kaplan–Meier curve shows the ability of *C. elegans* to survive after infection with ASBT-KP1. *E. coli* OP50 was the negative control. **(B)** Image of gravid adults scored as live when fed with *E. coli* OP50 (white arrow). **(C)** Live gravid adult worms infected with ASBT-KP1 (white arrow). **(D)** Worms scored as dead (red arrow). *p*-values ≥ 0.05 were considered non-significant from three independent experiments with replicates.

The negative result for the string test indicated a reduced virulent nature of ASBT-KP1. The *Caenorhabditis elegans* infected with ASBT-KP1 5 days post-infection were viable and healthy compared to *Escherichia coli* OP50-treated control (Figure 7A). The worms in the ASBT-KP1 infected wells showed active pharyngeal pumping and movement and were reproductively active and laid eggs (Figure 7C). The worms that were scored dead appeared rigid (Figure 7D) and did not show any movement compared to the *E. coli* OP50-treated worms (Figure 7B). Therefore, the strain is non-pathogenic in the surrogate worm model.

## 4 Discussion

Most of the culturable arsenic-resistant PGP bacteria are isolated from soil, the rhizosphere of mangroves, and industrial effluents (Butt and Rehman, 2011; Mesa et al., 2015; Mallick et al., 2018). Conversely, the arsenic-resistant PGP bacteria in the present study were isolated from domestic wastewater. Of the 11 isolates that grew on ASM plates, ASBT-KP1 and KP_PP2_2016 were the most promising. ASBT-KP1 was selected for further studies based on comparative PGP characteristics (Table 1) and high arsenic resistance (Figures 4A,B).

Over the years, different PGP rhizobacteria have been studied as potential candidates for improving plant growth quality and controlling diseases directly or indirectly. Many bacterial genera, including *Enterobacter* spp., *Klebsiella* spp., *Pseudomonas* spp., *Burkholderia* spp., *Bacillus* spp., and *Serratia* spp., have been identified for their potential to act as PGP rhizobacteria (Kim et al., 2022).

ASBT-KP1 was identified as *Klebsiella pneuominae* based on the whole genome sequence analysis. Tolerance of ASBT-KP1 to a pH range of 5–9, temperature resistance of 4–50°C (Figures 1B,C) and its ability to grow in high saline conditions make it an ideal candidate for operation in extreme soil conditions.

Most PGP bacteria are IAA and ACC deaminase producers and phosphate solubilisers that facilitate the absorption of nutrients from the soil, which promotes plant growth. Phosphorus is an essential nutrient for plants, and its availability is limited (1 μmol/L or less) by its existence primarily as an insoluble form in the soil. Hence, one of the essential functions of PGP bacteria is to solubilize mineral phosphate to soluble phosphorus and make it available for plants (Sashidhar and Podile, 2010). PGP bacteria stimulate plant growth directly by synthesizing the hormone IAA, which controls many physiological functions such as tissue differentiation, root initiation, cell enlargement and division, and phototropism (Leveau and Lindow, 2005). Organic acid production, such as HCN, implicated in protection against phytopathogens and hastening phosphate solubilization, is also an ideal feature (Rijavec and Lapanje, 2016). ASBT-KP1 possessed these favorable features of phosphate solubilization, IAA, and organic acids production (Table 1).

Whole genome sequence analysis consolidated these laboratory observations as the genes attributed to the IAA production, phosphate solubilization, and HCN production were identified in the genome of the ASBT-KP1. In addition, other PGP trait coding genes, including siderophore synthesis, acetoin, 2,3-butanediol synthesis, and those that confer fitness, were also identified. Similar findings were also reported in the species of *Klebsiella* (Liu et al., 2016) isolated from the rhizosphere soil of *Festuca arundinacea* L. PGP bacteria are also known to indirectly support plant growth by suppressing pathogenesis by producing antimicrobial compounds like phenazine, chitinase, and – ɣ aminobutyric acid. A similar profile is seen in the genome of ASBT-KP1, lending credence to its potential as a PGP bacterium (Supplementary Table S2). The genome of ASBT-KP1 also encodes enzymes such as superoxide dismutase, peroxidases, and glutathione transferases, which are known to suppress oxidative stress in plants. Comparative genomics analysis of ASBT-KP1 with *Klebsiella* spp. D5A strain and other related *Klebsiella* strains (Liu et al., 2016) establish its PGPR potential.

Similarly, ASBT-KP1 compares well with Klebsiella D5A regarding IAA production, phosphate solubilization, acetoin and 2,3-butanediol synthesis, and production of antimicrobial compounds. The genes involved in the survival of microorganisms under saline and temperature-stressed conditions were also detected in the ASBT-KP1 genome. Apart from these genes, the ASBT-KP1 genome also has acriflavin resistance protein A (acrA), stress response genes induced in bacteria upon plant colonization. AcrAB-TolC is an efflux pump that plays a vital role in removing toxic waste and the extrusion of drugs (Fouts et al., 2008). Under stressed conditions, these genes are activated and show an increased expression that could serve as a strategy to survive in arsenic-stressed conditions, marked by its ability to tolerate 120 mM of arsenate (Figure 4A,B). Another factor contributing to its tolerance toward high concentrations of arsenate is its ability to form biofilm. When compared to *Kocuria flava* and *Bacillus vietnamensis*, PGPBs with arsenic bioremediation capability (Mallick et al., 2018), ASBT-KP1 could also form a strong biofilm at a concentration of 50 mM of As (V). Biofilm and planktonic cells have varying degrees of tolerance to heavy metals, as biofilm helps sequestrate heavy metals, thus retarding the diffusion of heavy metals into the biofilm. Hence, biofilm formation is essential for heavy metal resistance (Harrison et al., 2004, 2007; Chien et al., 2013).

*Klebsiella* spp. are well-known arsenic-resistant bacteria that have been studied for their multi-heavy metal-resistant characteristics. RnASA11, a *Klebsiella pneumoniae* strain isolated from an arsenic-contaminated soil site, is one such isolate which could tolerate up to 600 mM of arsenate (V) (Kumar et al., 2021). To the best of our knowledge, *Klebsiella pneumonia* isolated from wastewater with a tolerance level of 120 mM toward arsenate (V) and 70 mM toward arsenite (III) has not been reported previously. ASBT-KP1 is thus an environmental isolate showing similarity to established *Klebsiella* PGP rhizobacteria. The strain’s antibiotic resistance (3 out of 16 antibiotics representing different classes) mechanism could be attributed to the resistance ASBT-KP1 shows against heavy metals and toxic metabolites. Although genes for virulence were predicted, pathogenicity testing in the *C. elegans* model yielded non-infectivity (Figure 7A). It is becoming highly imperative that the apprehensions about using apparent human pathogenic organisms as PGPR must be overcome to tap the immense potential to promote plant growth in polluted and stressed environmental conditions.

Promoting plant growth and reducing heavy metal toxicity and bioaccumulation in plants are research areas of focus in developing an eco-friendly and cost-effective strategy for sustainable agriculture (Mallick et al., 2018). Xenobiotics, like heavy metals, pesticides and similar other emerging pollutants, are likely to create physiological stress on the biological system (Mishra et al., 2021). Hence, exploring the potential microbes and plants that can effectively combat stress is paramount. Several bacterial isolates with multi-heavy metal resistance and varied salt tolerance have been studied for their potential as PGP bacteria and bio-remedial properties. *Klebsiella* spp. isolated from wastewater and soil alike has been extensively studied for its salt and heavy metal tolerance. In a study by Sapre et al. (2018), *Klebsiella* spp. isolated from the roots of wheat plants showed an enhancement in the growth of *Avena sativa* plants under heightened salinity conditions. In another study, *K. pneumoniae* and *K. variicola* isolated from industrial effluent were studied and employed to bioremediate arsenic-containing wastes (Butt and Rehman, 2011). Both, *K. pneumoniae* and *K. variicola* showed tolerance with a MIC of 26.6 and 24 mM, respectively, toward arsenic (Butt and Rehman, 2011). In comparison, ASBT-KP1 isolated from domestic wastewater tolerated up to 120 mM. Similarly, the phosphate solubilizing – 120 μg/mL and IAA production capacity – 210 mM, of the strain ASBT-KP1 in comparison to other known PGPBs, namely *Brevundimonas diminuta* (Singh et al., 2016), *Pseudomonas mosselii* and *Bacillus thuringiensis* (Aw et al., 2020) (phosphate solubilization – 96 μg/mL, 1.32 mg/L and 1.30 mg/mL; 57 μg/mg, 6.36 μg/mL and 14.95 μg/mL respectively) is higher. Thus, the findings of this study are consistent with published literature and indicate the potential of ASBT-KP1 as a rhizoinoculant.

Finally, the study establishes the role of ASBT-KP1 in alleviating arsenic stress in *V. radiata.* Inoculation with ASBT-KP1 improved the plant growth function and the dry biomass and chlorophyll content in the presence of arsenic. The decrement in plant shoot and root length in uninoculated *V. radiata* might be due to the toxic effect of arsenic on plants. The influence includes modification of oxidative stress, inactivation of metabolic enzymes and proteins due to their affinity to sulfhydryl groups, and localization in roots leading to disruption in symbiotic N_2_ fixation and assimilation (Finnegan and Chen, 2012). A 91% reduction in arsenic plant bioaccumulation (shoot) was observed on treatment with the PGP bacteria (Figure 6F). At the shoot and root length level, a significant difference is seen in the presence and absence of ASBT-KP1 (Figures 6B,C). Similar enhancement is also reflected in total plant biomass (Figure 6D). These establish the growth promotion potential of ASBT-KP1. The productivity also increased significantly which is indicated by the difference in total chlorophyll content of the ASBT-KP1-inoculated plants when compared to control (Figure 6E). It is also interesting to note that these parameters did not vary significantly when the stressed, inoculated plants were compared with their stress-free inoculated counterparts. Though shoot and root length showed a marginal decrease of 0.09 and 0.36 cm, respectively, plant biomass and chlorophyll content, on the contrary, showed an increase of 7.75 and 17.2%, respectively. Active absorption of heavy metal by ASBT-KP1 consolidates its role in relieving the arsenic stress of the associated plants apart from improving other plant growth parameters. Abatement of arsenic uptake in the plants might be due to the biofilm-forming ability and arsenic resistance genes. The strain on its introduction into the rhizospheric soil was effectively established since its presence at a density of 1.4 × 10^5^ CFU/mL was detected after 8 days against an almost uniform microbial backdrop (control – 3.5 × 10^6^ CFU/mL; test – 5.5 × 10^6^ CFU/mL).

Moreover, traces of the strain were not detected in the shoot and leaves, but on the contrary, the root showed 8.2 × 10^3^ CFU/mL, which is 2 log less than the density found in the rhizospheric soil. These consolidate its potential in field applications and the strain’s useful properties. Thus, the soil bioaugmentation with ASBT-KP1 contributed to plant growth promotion while protecting the plant from the adverse effects of arsenic stress (Figure 6A). ASBT-KP1 could also find application in other plants as well. Recent research demonstrates that inoculating rice plants with rhizoinoculants possessing plant growth promotion (PGP) and heavy metal bioremediation capabilities can enhance plant growth and mitigate arsenic accumulation in plant tissues. Preliminary studies on *O. sativa* have proven to be promising with enhancement in PGP and arsenic tolerance potential (Supplementary Figure S4). ASBT-KP1 decreased the arsenic accumulation in *O. sativa* by 74%. These results align with the earlier *O. sativa* studies, demonstrating that heavy metal-tolerant bacteria can reduce metal uptake by host plants (Singh et al., 2016; Aw et al., 2020).

The described study demands an extensive assessment in the field conditions to analyze various drivers including environmental and biological factors that influence host–microbe interactions in the contaminated soil. The strain’s stability, composition and bioactivity need to be fully explored for effective application aimed at improving plant growth and productivity. Further studies can be carried out as a follow-up to the described one, to factor in the role of rhizosphere microorganisms and the microorganisms symbiotically associated with the roots. Apart from its projected application in agriculture, it could potentially be used in microbial bioremediation in conjunction with the phytoremediation of wastewater, which might prove useful in fertigation (fertilization and irrigation). Moreover, ASBT-KP1 could be potentially used in biofilters associated with vertical and hydroponic systems to remove heavy metal contaminants and improve productivity (in the case of vertical gardens).

## 5 Conclusion

The present study identifies ASBT-KP1 as a potential PGP bacteria efficient in arsenic (V) absorption, and promotion of the growth of *V. radiata.* The strain effectively absorbed arsenic and tolerated up to a concentration of 120 mM (Na_2_HAsO_4_. 7H_2_O). ASBT-KP1 was identified as a *K. pneumoniae*, and its sequence is deposited in NCBI. Biochemical profiling of the isolate showed that it could produce ammonia, IAA, ACC deaminase and HCN and could solubilize phosphate. Experiments with *V. radiata* in arsenic-contaminated soil showed a concurrent plant growth promotion, a significant (*p* < 0.05) increase in plant biomass, length, and chlorophyll content, and reduced uptake of arsenic in *V. radiata* plants inoculated with ASBT-KP1. The study was replicated in *O. sativa* and concurrent results were observed, which demonstrates the potential of ASBT-KP1 in non-leguminous plant. The presence of *arsCBR* genes is attributed to the significant resistance toward arsenate and arsenite (120 mM and 70 mM, respectively). ASBT-KP1-treated *V. radiata* plants showed increased plant biomass and chlorophyll compared to the control. Plants grown in the presence of ASBT-KP1 also showed a 91% reduced accumulation of arsenic compared to the control. The successful inoculation of ASBT-KP1 to the plant rhizosphere could help alleviate arsenic toxicity in *V. radiata* plants grown in arsenic-contaminated soil and be used as a potential mitigation strategy for the remediation of arsenic from soil.

## Data Availability

The datasets presented in this study can be found in online repositories. The names of the repository/repositories and accession number(s) can be found in the article/Supplementary material.

## Footnotes

^1^http://www.ncbi.nlm.nih.gov/
