# Supplementary material for: Alleviating arsenic stress affecting the growth of Vigna radiata through the application of Klebsiella strain ASBT-KP1 isolated from wastewater
Source: Front Microbiol. 2024 Sep 25;15:1484069. doi: 10.3389/fmicb.2024.1484069 (PMC11461332; doi:10.3389/fmicb.2024.1484069)
Supplement: Supplementary file 1 [file Data_Sheet_1.PDF]

```
# ResFinder phenotype results.
# Sample: MTB_assembly_contigs.fasta
#
# The phenotype 'No resistance' should be interpreted with
# caution, as it only means that nothing in the used
# database indicate resistance, but resistance could exist
# from 'unknown' or not yet implemented sources.
#
# The 'Match' column stores one of the integers 0, 1, 2, 3.
#      0: No match found
#      1: Match < 100% ID AND match length < ref length
#      2: Match = 100% ID AND match length < ref length
#      3: Match = 100% ID AND match length = ref length
# If several hits causing the same resistance are found,
# the highest number will be stored in the 'Match' column.
```

# Antimicrobial	Class	WGS-predicted phenotype	Match	Genetic background
metronidazole	nitroimidazole	No resistance	0	
mupirocin	pseudomonic acid	No resistance	0	
teicoplanin	glycopeptide	No resistance	0	
vancomycin	glycopeptide	No resistance	0	
penicillin	beta-lactam	No resistance	0	
temocillin	beta-lactam	No resistance	0	
piperacillin+tazobactam	beta-lactam	No resistance	0	
ampicillin	beta-lactam	No resistance	0	
cefepime	beta-lactam	No resistance	0	
ceftazidime	beta-lactam	No resistance	0	
ceftriaxone	beta-lactam	No resistance	0	
unknown beta-lactam	beta-lactam	No resistance	0	
amoxicillin+clavulanic acid	beta-lactam	No resistance	0	
cephalotin	beta-lactam	No resistance	0	
ceftazidime+avibactam	beta-lactam	No resistance	0	
aztreonam	beta-lactam	No resistance	0	
piperacillin	beta-lactam	No resistance	0	
cephalothin	beta-lactam	No resistance	0	
amoxicillin	beta-lactam	No resistance	0	
cefotaxime	beta-lactam	No resistance	0	
cefixime	beta-lactam	No resistance	0	
ticarcillin	beta-lactam	No resistance	0	
cefoxitin	beta-lactam	No resistance	0	
piperacillin+clavulanic acid	beta-lactam	No resistance	0	
ampicillin+clavulanic acid	beta-lactam	No resistance	0	
cefotaxime+clavulanic acid	beta-lactam	No resistance	0	
ertapenem	beta-lactam	No resistance	0	
imipenem	beta-lactam	No resistance	0	
ticarcillin+clavulanic acid	beta-lactam	No resistance	0	
meropenem	beta-lactam	No resistance	0	
spectinomycin	aminocyclitol	No resistance	0	
oleandomycin	macrolide	No resistance	0	
erythromycin	macrolide	No resistance	0	
tylosin	macrolide	No resistance	0	
azithromycin	macrolide	No resistance	0	
spiramycin	macrolide	No resistance	0	

telithromycin	macrolide	No resistance	0
carbomycin	macrolide	No resistance	0
formaldehyde	aldehyde	No resistance	0
temperature	heat	No resistance	0
chlorhexidine	quaternary ammonium compound	No resistance	0
cetylpyridinium chloride	quaternary ammonium compound	No resistance	0
benzylkonium chloride	quaternary ammonium compound	No resistance	0
ethidium bromide	quaternary ammonium compound	No resistance	0
hydrogen peroxide	peroxide	No resistance	0
tetracycline	tetracycline	No resistance	0
tigecycline	tetracycline	No resistance	0
doxycycline	tetracycline	No resistance	0
minocycline	tetracycline	No resistance	0
tiamulin	pleuromutilin	No resistance	0
fluoroquinolone	quinolone	No resistance	0
nalidixic acid	quinolone	No resistance	0
unknown quinolone	quinolone	No resistance	0
ciprofloxacin	quinolone	No resistance	0
florfenicol	amphenicol	No resistance	0
chloramphenicol	amphenicol	No resistance	0
rifampicin	rifamycin	No resistance	0
trimethoprim	folate pathway antagonist	No resistance	0
sulfamethoxazole	folate pathway antagonist	No resistance	0
hygromycin	aminoglycoside	No resistance	0
unknown aminoglycoside	aminoglycoside	No resistance	0
streptomycin	aminoglycoside	No resistance	0
bleomycin	aminoglycoside	No resistance	0
amikacin	aminoglycoside	No resistance	0
paromomycin	aminoglycoside	No resistance	0
dibekacin	aminoglycoside	No resistance	0
apramycin	aminoglycoside	No resistance	0
astromicin	aminoglycoside	No resistance	0
isepamicin	aminoglycoside	No resistance	0
gentamicin	aminoglycoside	No resistance	0
sisomicin	aminoglycoside	No resistance	0
neomycin	aminoglycoside	No resistance	0
ribostamycin	aminoglycoside	No resistance	0
butiromycin	aminoglycoside	No resistance	0
tobramycin	aminoglycoside	No resistance	0
arbekacin	aminoglycoside	No resistance	0
butirosin	aminoglycoside	No resistance	0
lividomycin	aminoglycoside	No resistance	0
kasugamycin	aminoglycoside	No resistance	0
fortimicin	aminoglycoside	No resistance	0
netilmicin	aminoglycoside	No resistance	0
kanamycin	aminoglycoside	No resistance	0
colistin	polymyxin	No resistance	0
fusidic acid	steroid antibacterial	No resistance	0
pristinamycin ia	streptogramin b	No resistance	0
virginiamycin s	streptogramin b	No resistance	0
quinupristin	streptogramin b	No resistance	0
dalfopristin	streptogramin a	No resistance	0

virginiamycin m	streptogramin a	No resistance	0
pristinamycin iia	streptogramin a	No resistance	0
quinupristin+dalfopristin	streptogramin a	No resistance	0
fosfomycin	fosfomycin	No resistance	0
linezolid	oxazolidinone	No resistance	0
ceftiofur	under_development	No resistance	0
cephalosporins	under_development	No resistance	0
norfloxacin	under_development	No resistance	0
carbapenem	under_development	No resistance	0
lincomycin	lincosamide	No resistance	0
clindamycin	lincosamide	No resistance	0
