# Supplementary material for: Alleviating arsenic stress affecting the growth of Vigna radiata through the application of Klebsiella strain ASBT-KP1 isolated from wastewater
Source: Front Microbiol. 2024 Sep 25;15:1484069. doi: 10.3389/fmicb.2024.1484069 (PMC11461332; doi:10.3389/fmicb.2024.1484069)
Supplement: Supplementary file 2 [file Data_Sheet_2.pdf]

Alleviating arsenic stress affecting the growth of *Vigna radiata* through the application of *Klebsiella* strain ASBT-KP1 isolated from wastewater

Megha Prasad[1], Ajith Madhavan[1*], Pradeesh Babu[1], Amrita Salim[1], Suja Subhash[1], Bipin G Nair[1], Sanjay Pal[1*]
Affiliation 1. School of Biotechnology, Amrita Vishwa Vidyapeetham, Kerala, 690525, India

[*]Dr Sanjay Pal, sanjaypal@am.amrita.edu
[*]Dr Ajith Madhavan, ajithm@am.amrita.edu

Serial no.	Test	ASBT-KP1
1	Lactose	+
2	Xylose	+
3	Maltose	+
4	Fructose	+
5	Dextrose	+
6	Galactose	+
7	Raffinose	+
8	Trehalose	+
9	Melibiose	+
10	Sucrose	+
11	L-Arabinose	+
12	Mannose	+
13	Inulin	-
14	Sodium gluconate	-
15	Glycerol	-
16	Salicin	+
17	Dulcitol	-
18	Inositol	+
19	Sorbitol	+
20	Mannitol	+
21	Adonitol	+
22	Arabitol	+
23	Erythritol	-
24	α-Methyl-D-glucoside	-
25	Rhamnose	+
26	Cellobiose	+
27	Melezitose	-
28	α-Methyl-D-mannoside	+
29	Xylitol	-

30	ONPG	+
31	Esculin hydrolysis	+
32	D-Arabinose	-
33	Citrate utilisation	+
34	Malonate utilisation	+
35	Sorbose	-
36	Control	

Table S1 Carbohydrate utilisation profile of the isolate ASBT-KP1

Function	Gene location	Gene annotation	KEGG_GENE_NAME
Indole-3-acetic acid biosynthesis	spades_contig_8_117010+1440	Aldehyde dehydrogenase A@ Glycolaldehyde dehydrogenase	EC 1.2.1.22 EC 1.2.1.21
	spades_contig_3_287385-789	Aliphatic amidase AmiE	EC 3.5.1.4
	spades_contig_5_312222+786	Aliphatic amidase AmiE	EC 3.5.1.4
Phosphate solubilisation	spades_contig_4_513+1029	Quinate/shikimate dehydrogenase [Pyrroloquinoline-quinone]	EC 1.1.5.8
	spades_contig_1_410152-756	Pyrroloquinoline-quinone synthase	EC 1.3.3.11
	spades_contig_18_89729+2391	Glucose dehydrogenase, PQQ-dependent	EC 1.1.5.2
	spades_contig_1_407987-2286	Coenzyme PQQ synthesis protein F	pqqF
	spades_contig_1_409129-1143	Coenzyme PQQ synthesis protein E	pqqE
	spades_contig_1_409394-279	Coenzyme PQQ synthesis protein D	pqqD
	spades_contig_1_410152-756	Pyrroloquinoline-quinone synthase	EC 1.3.3.11
	spades_contig_1_411072-927	Coenzyme PQQ synthesis protein B	pqqB
Phosphate uptake	spades_contig_17_30653+1041	Phosphate ABC transporter, substrate-binding protein PstS	PstS (TC 3.A.1.7.1)
	spades_contig_17_31822+960	Phosphate ABC transporter, permease protein PstC	PstC (TC 3.A.1.7.1)
	spades_contig_17_32781+891	Phosphate ABC transporter, permease protein PstA	PstA (TC 3.A.1.7.1)
	spades_contig_17_33719+774	Phosphate ABC transporter, ATP-binding protein PstB	PstB (TC 3.A.1.7.1)
	spades_contig_6_141920+1497	Low-affinity inorganic phosphate transporter	
	spades_contig_2_497159+801	ABC-type phosphate/phosphonate transport system, periplasmic component	
	spades_contig_5_246665+843	Phosphonate ABC transporter ATP-binding protein PhnC	PhnC (TC 3.A.1.9.1)
	spades_contig_5_247530+939	Phosphonate ABC transporter substrate-binding protein PhnD	PhnD (TC 3.A.1.9.1)
	spades_contig_5_248535+873	Phosphonate ABC transporter permease protein PhnE2	PhnE2 (TC 3.A.1.9.1)
	spades_contig_5_249404+885	Phosphonate ABC transporter permease protein PhnE1	PhnE1 (TC 3.A.1.9.1)

Siderophore production	spades_contig_3_346641-756	2,3-dihydro-2,3-dihydroxybenzoate dehydrogenase [enterobactin] siderophore	EC 1.3.1.28
	spades_contig_3_347492-852	Isochorismatase [enterobactin] siderophore / Apo-aryl carrier domain of EntB	EC 3.3.2.1
	spades_contig_3_349113-1608	2,3-dihydroxybenzoate-AMP ligase [enterobactin] siderophore	EC 2.7.7.58
	spades_contig_3_350298-1176	Isochorismate synthase	EC 5.4.4.2
	spades_contig_3_353217-1242	Enterobactin exporter EntS	EntS
	spades_contig_3_364068+630	4'-phosphopantetheinyl transferase [enterobactin] siderophore	
	spades_contig_3_360063-3882	Enterobactin synthetase component F, serine activating enzyme	
Fe uptake	spades_contig_10_121970-2259	TonB-dependent receptor; Outer membrane receptor for ferric enterobactin and colicins B, D	
	spades_contig_35_5822+2064	TonB-dependent receptor	
	spades_contig_1_306621-2106	Probable tonB-dependent receptor yncD precursor	
	spades_contig_3_361773+2229	TonB-dependent receptor; Outer membrane receptor for ferric enterobactin and colicins B, D	
	spades_contig_4_268751-2364	TonB-dependent hemin, ferrichrome receptor	
Fe transport	spades_contig_7_78864-1284	Ferrous iron transport peroxidase EfeB	EfeB
	spades_contig_7_79996-1128	Ferrous iron transport periplasmic protein EfeO	EfeO
	spades_contig_7_80878-831	Ferrous iron transport permease EfeU	EfeU
	spades_contig_9_28408-777	Iron-chelator utilisation protein	
	spades_contig_10_107486-858	ABC transporter, permease protein 2 (cluster 1, iron)	
	spades_contig_10_109516-1140, spades_contig_1_129175+1089, spades_contig_1_132099+1071, spades_contig_4_98099-1287	ABC transporter, substrate-binding protein (cluster 1, iron)	
	spades_contig_10_110628-1083, spades_contig_14_11041+1071, spades_contig_1_108175+774, spades_contig_1_132099+1071, spades_contig_4_99243-1128	ABC transporter, ATP-binding protein (cluster 1, iron)	

	spades_contig_19_48166+2286 spades_contig_1_111248-2073, spades_contig_2_489422-2121, spades_contig_3_444189-2232,	Ferrichrome-iron receptor	
	spades_contig_19_54155+2247	Outer Membrane Siderophore Receptor IroN	
	spades_contig_1_88123-750 spades_contig_3_467411+798	ABC transporter, ATP-binding protein (cluster 8, B12/iron complex)	
	spades_contig_1_89127-1008	ABC-type Fe3+-siderophore transport system, permease component	
	spades_contig_1_90175-1059	ABC transporter, substrate-binding protein (cluster 8, B12/iron complex)	
	spades_contig_35_18363-768	Iron(III) dicitrate transport ATP-binding protein	FecE
	spades_contig_35_19320-957 spades_contig_35_20315-999	Iron(III) dicitrate transport system permease protein	FecD, FecC
	spades_contig_35_21214-903	Iron(III) dicitrate transport system, periplasmic iron-binding protein	FecB
	spades_contig_35_23583-2325	Iron(III) dicitrate transport protein	FecA
	spades_contig_35_24623-954	Iron(III) dicitrate transmembrane sensor protein	FecR
	spades_contig_35_25141-522	RNA polymerase sigma factor	FecI
	spades_contig_1_111754+822	Iron compound ABC transporter, ATP-binding protein	
	spades_contig_1_112572+1026 spades_contig_1_113594+1053	Iron compound ABC transporter, permease protein	
	spades_contig_1_114660+954 spades_contig_3_465206+1119	ABC transporter, substrate-binding protein (cluster 8, B12/iron complex)	
	spades_contig_1_130337+1770		
	spades_contig_3_466332+1083	ABC transporter, permease protein (cluster 8, B12/iron complex)	
	spades_contig_4_100158-903	ABC transporter, permease protein 2 (cluster 1, iron)	
	spades_contig_4_101041-891	ABC transporter, permease protein 1 (cluster 1, iron)	

	spades_contig_5_57995-783	Probable iron export permease protein	FetB
	spades_contig_5_58659-678	Probable iron export ATP-binding protein	FetA
	spades_contig_6_54032+180<br>spades_contig_6_54231+228	Ferrous iron transporter-associated protein	FeoA
	spades_contig_6_54487+2319	Ferrous iron transporter	FeoB
	spades_contig_6_56815+240	Ferrous iron-sensing transcriptional regulator	FeoC
	spades_contig_16_47162-426	TonB-ExbBD energy transducing system	ExbD
	spades_contig_16_47900-732	TonB-ExbBD energy transducing system	ExbB
	spades_contig_1_22803+747	TonB-ExbBD energy transducing system	TonB
	spades_contig_4_276962-918	Iron/Manganese ABC transporter, periplasmic-binding protein	SitA
	spades_contig_4_274388-855	Iron/Manganese ABC transporter, inner membrane permease protein	SitD
	spades_contig_4_275230-849	Iron/Manganese ABC transporter, inner membrane permease protein	SitC
	spades_contig_4_276048-822	Manganese ABC transporter, ATP-binding protein	SitB
Heavy metal resistance	spades_contig_5_60686+2502<br>spades_contig_33_14146-2448<br>spades_contig_5_60686+2502<br>spades_contig_6_117765+2211	Lead, cadmium, zinc and mercury transporting ATPase; Copper-translocating P-type ATPase	EC 3.6.3.3<br>EC 3.6.3.5<br>EC 3.6.3.4
	spades_contig_1_257666+276	RcnR-like protein clustered with cobalt-zinc-cadmium resistance protein CzcD	CzcD
	spades_contig_1_388480+924	Cobalt-zinc-cadmium resistance protein	
	spades_contig_5_60579-411	Copper resistance transcriptional regulator CueR (MerR family)	CueR
	spades_contig_2_431448+375	Copper resistance protein CopC	CopC
	spades_contig_2_431826+870	Copper resistance protein CopD	CopD
	spades_contig_3_260717+1539	Apolipoprotein N-acyltransferase / Copper homeostasis protein CutE	CutE

	spades_contig_4_22136-504	Membrane protein, suppressor for copper-sensitivity ScsD	ScsD
	spades_contig_4_22737-612	Secreted protein, suppressor for copper-sensitivity ScsC	ScsC
	spades_contig_4_24748-2010	Membrane protein, suppressor for copper-sensitivity ScsB	ScsB
	spades_contig_4_25157-360	Suppression of copper sensitivity: putative copper binding protein ScsA	ScsA
	spades_contig_4_47801+330	Arsenical resistance operon repressor	ArsR
	spades_contig_4_48182+1293	Arsenite/antimonite:H+ antiporter ArsB	ArsB
	spades_contig_4_49484+423	Arsenate reductase glutaredoxin-coupled, glutaredoxin-like family	ArsC/EC 1.20.4.1
Resistance to oxidative stress			
Superoxide dismutase	spades_contig_1_221716-582	Superoxide dismutase [Fe]	EC 1.15.1.1
	spades_contig_1_228652+522	Superoxide dismutase [Cu-Zn] precursor	EC 1.15.1.1
	spades_contig_20_49145+621	Superoxide dismutase [Mn]	EC 1.15.1.1
Glutathione S-transferase	spades_contig_1_240522-606 spades_contig_2_57096-630	Glutathione S-transferase	EC 2.5.1.18
	spades_contig_1_257966+1119	S-(hydroxymethyl) glutathione dehydrogenase	EC 1.1.1.284
	spades_contig_1_319423+618	Glutathione S-transferase, theta	EC 2.5.1.18
	spades_contig_2_57277+639	Probable glutathione S-transferase, YfcF homolog	EC 2.5.1.18
Peroxidase	spades_contig_12_111250-471	Thiol peroxidase, Bcp-type	EC 1.11.1.15
	spades_contig_12_152410+900	Predicted dye-decolorising peroxidase (DyP), YfeX-like subgroup	
	spades_contig_24_24935+483	Glutathione peroxidase	EC 1.11.1.9
	spades_contig_25_22194-507	Thiol peroxidase, Tpx-type	EC 1.11.1.15
	spades_contig_1_41618+552	Glutathione peroxidase	EC 1.11.1.9
	spades_contig_1_400525-2178	Catalase-peroxidase KatG	EC 1.11.1.21
Rhodanese: catalysis of the detoxification of cyanide	spades_contig_7_59382-1065	Rhodanese domain protein, Enterobacterial subgroup, YceA homolog	
	spades_contig_13_112365+432	Rhodanese-related sulfurtransferase YibN	YibN
	spades_contig_19_85985+1308	Thiosulfate sulfurtransferase, rhodanese	EC 2.8.1.1

	spades_contig_23_25979-546	Rhodanese-domain-containing inner membrane protein YgaP	YgaP
	spades_contig_1_101217-1581	Rhodanese-related sulfurtransferase, 4 domains	
Antimicrobial compounds			
Phenazine production	spades_contig_1_604293-795	Phenazine biosynthesis protein	PhzF
Chitinase production	spades_contig_24_13814+1254	Chitinase	EC 3.2.1.14
ɣ-aminobutyric acid (GABA)	spades_contig_1_593315-1389 spades_contig_22_52800-1371	Succinate-semialdehyde dehydrogenase [NAD]; Succinate-semialdehyde dehydrogenase [NADP+]	EC 1.2.1.24 EC 1.2.1.79
	spades_contig_5_288875+1449	Succinate-semialdehyde dehydrogenase [NAD(P)+]	EC 1.2.1.16
	spades_contig_5_290336+1284	Gamma-aminobutyrate:alpha-ketoglutarate aminotransferase	EC 2.6.1.19
	spades_contig_5_291769+1401	gamma-aminobutyrate (GABA) permease	
	spades_contig_6_109440-1266	Gamma-aminobutyrate:alpha-ketoglutarate aminotransferase	EC 2.6.1.19
	spades_contig_6_109559+1524	Transcriptional regulator GabR of GABA utilisation	GabR
Acetoin and 2, 3-butanediol synthesis	spades_contig_1_158115-1680	Acetolactate synthase, catabolic	EC 2.2.1.6
	spades_contig_1_158904-780	Alpha-acetolactate decarboxylase	EC 4.1.1.5
	spades_contig_1_156412-771	2,3-butanediol dehydrogenase, S-alcohol forming, (R)-acetoin-specific / Acetoin (diacetyl) reductase	EC 1.1.1.4/EC 1.1.1.304
	spades_contig_11_54470-522, spades_contig_17_85419+288, spades_contig_21_87507-258	Acetolactate synthase small subunit	EC 2.2.1.6
	spades_contig_11_56167-1725, spades_contig_17_83727+1689, spades_contig_21_89150-1647	Acetolactate synthase large subunit	EC 2.2.1.6

Table S2 Genes related to PGP traits

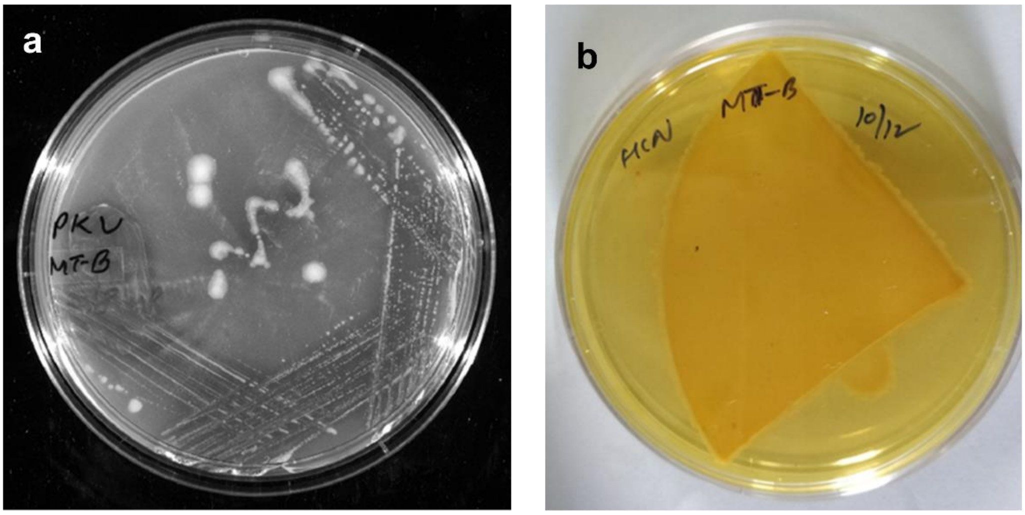

Fig S1 Plant growth promoting characteristics of ASBT-KP1. The image shows a) the phosphate solubilisation of inorganic phosphate by the strain ASBT-KP1. Image b) shows the ability of the strain to produce HCN.

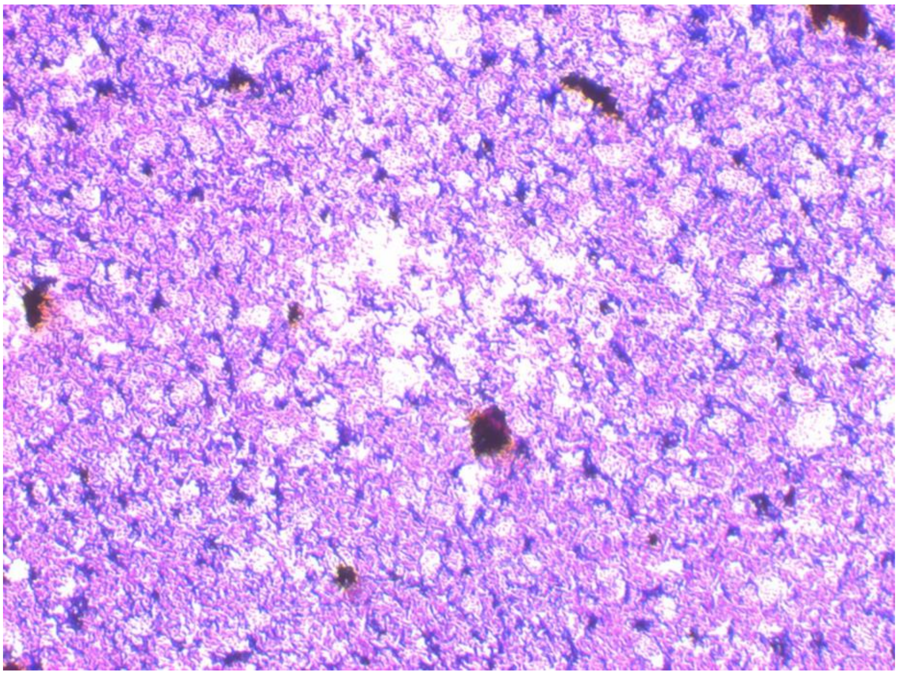

Fig S2 Biofilm formation**.** ASBT-KP1 was cultured in R2-A broth supplemented with arsenate in vitro for 18 hours in 24-well plates at 37 °C and stained with crystal violet (100 X) for imaging the biofilm.

Property	Quantity
Texture	Coarse textured
Phosphate, mg/Kg	16.7
Total Kjeldahl nitrogen, %	0.14
Ammonia, mg/Kg	154
Arsenic, mg/Kg	< 0.1
Total organic carbon, %	0.78
Manganese, ppm	1.13
Iron, ppm	26
Zinc, ppm	1.7
Copper, ppm	1.2
pH	5.5
Total suspended solids, mhos/cm	0.10

Table S3 Physicochemical properties of soil

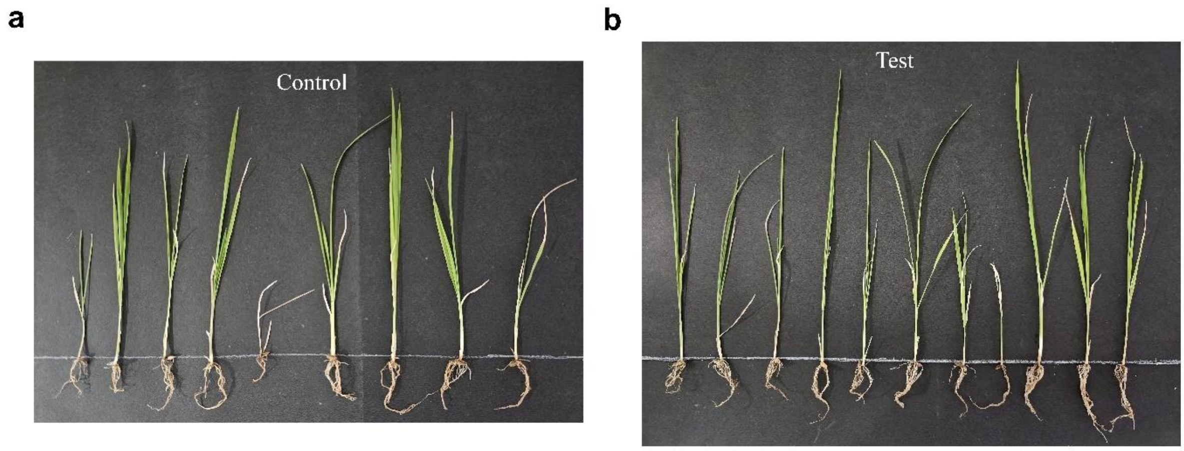

Fig S3: A pictorial comparison of the *O. sativa* grown in unsterile soil a) without arsenate and b) with arsenate.

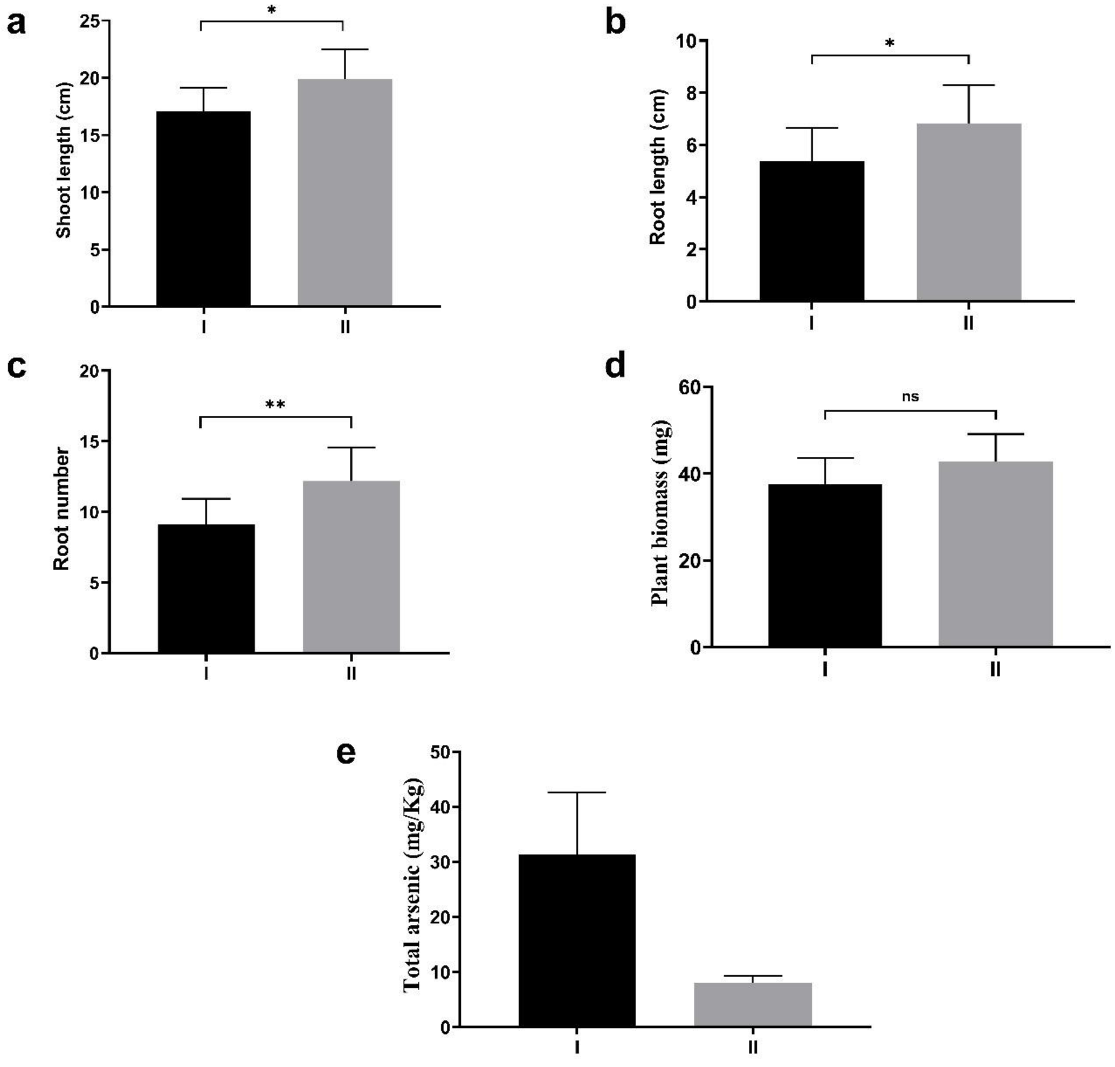

Fig S4: Effect of ASBT-KP1 on *O. sativa* under arsenic stress. Estimation of different plant growth-promoting properties of ASBT-KP1 in *O. sativa* under As (V) stress a) shoot length; b) root length; c) root number; d) plant biomass, and e) As accumulation in the *O. sativa* treated with ASBT-KP1 compared to control sets without any inoculation. [I – Plant + As and II – Plant + As + ASBT-KP1].
